# Status epilepticus-induced 12/15-lipoxygenase drives neuroinflammation and contributes to neuronal injuries and behavioral comorbidities

**DOI:** 10.1038/s41401-025-01743-z

**Published:** 2026-02-13

**Authors:** Md. Asaduzzaman Rakib, Eun Bee Cho, Nelufar Yasmen, Chenyao Jiang, Md. Aktaruzzaman, Juqian Han, Ying Yu, Jianxiong Jiang

**Affiliations:** 1 Department of Pharmaceutical Sciences, College of Pharmacy, The University of Tennessee Health Science Center, Memphis, TN, USA; 2 Center for Pediatric Neurological Disease Research, St. Jude Children’s Research Hospital, Memphis, TN, USA

**Keywords:** 12/15-LOX, epilepsy, ML351, neurobehavioral comorbidities, neuroinflammation, seizure

## Abstract

Status epilepticus (SE) is a serious neurological condition defined as a continuous seizure lasting longer than 5 min or multiple seizures without full recovery of consciousness between them. Uncontrolled SE causes severe brain inflammation and damage, leading to life-long epilepsy and behavioral comorbidities. 12/15-Lipoxygenase (12/15-LOX), an enzyme that generates bioactive lipid metabolites from polyunsaturated fatty acids, plays pathogenic role in oxidative and inflammatory processes that can aggravate tissue injuries. However, its involvement in SE-triggered neuroinflammation and long-term sequelae remains elusive. Herein, we report that 12/15-LOX was significantly upregulated in microglia in response to inflammatory stimuli as well as in the hippocampus after pilocarpine-induced SE in mice. Selective inhibition of 12/15-LOX by compound ML351 robustly reduced lipopolysaccharide-provoked pro-inflammatory gene expression both in vitro and in vivo. Treatment with ML351 (50 mg/kg, i.p.) after SE was interrupted by diazepam markedly decreased pro-inflammatory cytokines and reactive gliosis and broadly prevented neuronal injuries within the hippocampus. Moreover, repeated administration of ML351 for merely five consecutive days after SE led to a long-term improvement in spatial working and reference memory along with a reduction in anxiety-like behavior as well as an increase in hippocampal neuronal survival. These results suggest that inhibition of 12/15-LOX hours after SE onset can alleviate neuroinflammation, protect hippocampal neurons, and prevent long-term neurobehavioral deficits. Therefore, targeting 12/15-LOX might provide an adjunctive strategy, together with the current antiseizure medications, to mitigate neurobehavioral comorbidities associated with prolonged seizures.

## INTRODUCTION

Status epilepticus (SE) is the second most common life-threatening neurological emergency. Annually, up to 41 cases per 100,000 individuals are reported, with an overall mortality rate of approximately 32% [1]. Considering the resolution of the destructive effect of seizures, clinical definitions and treatment guidelines emphasize the urgency of early intervention with antiseizure medications (ASMs) to prevent irreversible brain damage [2, 3]. However, current ASMs often fail to control seizures in a significant subset of patients and are also associated with adverse effects that limit their long-term use [4, 5]. SE is a common consequence of other brain conditions, such as strokes, central nervous system (CNS) infections, head injuries, encephalitis, brain tumors, genetic defects, and intoxications [6]. Regardless of etiology, neuroinflammation has emerged as a central mechanism in the pathophysiology of SE and its aftermath. It is thought to form a vicious cycle where SE initiates inflammatory processes, which in turn exacerbate brain damage, lower seizure threshold, trigger epileptogenesis [7], and eventually lead to life-long epilepsy and the associated behavioral comorbidity conditions, such as anxiety, depression, and cognitive disabilities [8, 9].

Among the key biochemical pathways involved in neuroinflammation is the metabolism of arachidonic acid (AA). Following an episode of SE, free AA is released into the cytoplasm from the phospholipid bilayer and is metabolized via three pathways governed by cyclooxygenases (COXs), lipoxygenases (LOXs), and cytochrome P450 (CYP) enzymes. While the COX pathway has been extensively studied as a possible target to prevent SE-triggered neuropathogenesis, many COX inhibitors showed both beneficial and detrimental effects in preclinical models, suggesting its dual roles in different phases of inflammation after SE [10, 11]. In addition, COX inhibition is commonly associated with increased risk of developing severe cardiovascular conditions, such as stroke and myocardial infarction [12]. As such, the LOX pathway has recently gained considerable attention as an emerging alternative target. Among the currently known LOX isoforms, 12/15-LOX (known as 15-LOX-1 in human) is widely expressed throughout the CNS and thus is often referred to as the neuronal isoform of LOX [13, 14]. It specifically inserts oxygens into AA at the C-12/15 position to generate 12-HpETE and 15-HpETE, which are further converted into more stable 12-HETE and 15-HETE [15]. These active metabolites can bind to peroxisome proliferator-activated receptor γ (PPAR-γ) and trigger production of reactive oxygen species (ROS) [16], which can lead to inflammatory responses and contribute to neuronal damage via oxidizing cellular components. Therefore, 12/15-LOX is known for a pathophysiological role in inflammation-related CNS diseases [17], and indeed, its inhibition has demonstrated promising neuroprotective effects in models of stroke [18-20]. Despite the growing body of evidence supporting an essential role of 12/15-LOX in various neurological conditions, its contribution to prolonged seizure-induced neuroinflammation and long-term neuropathogenesis remains unexplored.

The goal of this study was to elucidate the role of 12/15-LOX in both acute and chronic phases of SE-induced brain inflammation and pathologies. Utilizing mouse models of lipopolysaccharide (LPS)-provoked neuroinflammation and pilocarpine-induced SE, we examined the effects of selective 12/15-LOX inhibition with ML351 on inflammatory gene expression, neuronal survival, glial activation, and long-term behavioral outcomes. The present work revealed that 12/15-LOX upregulation was associated with the initiation of neuroinflammatory responses and identified 12/15-LOX as a feasible anti-inflammatory target in a preclinical model of SE. Importantly, 12/15-LOX blockade along with ASMs demonstrated therapeutic potential to mitigate both immediate and delayed consequences of SE.

## MATERIALS AND METHODS

### Chemicals and drugs

Methylscopolamine, terbutaline, pilocarpine, LPS, and Fluoro-Jade B were purchased from Sigma-Aldrich (Burlington, MA, USA). Parenteral diazepam (5 mg/mL, Hospira) was purchased from Henry Schein (Melville, NY, USA). Compound ML351 [5-(methylamino)-2-(naphthalen-1-yl)oxazole-4-carbonitrile] was purchased from Combi-Blocks (San Diego, CA, USA) and authenticated using ^1^H NMR (Supplementary Fig. S1) and HRMS (Supplementary Fig. S2), and the purity was 97.44%.

### Animals

Adult male C57BL/6 mice (about 8 weeks old and 22–25 g) were purchased from Charles River Laboratories and housed and maintained at room temperature in standard humidity conditions under 12-h light/dark cycle with free access to food and water. All animal procedures were approved by the Institutional Animal Care and Use Committee (IACUC) of the University of Tennessee Health Science Center (Protocol # 24-0551). All experiments were performed in accordance with the Guide for the Care and Use of Laboratory Animals (the Guide) and reported according to ARRIVE Guidelines 2.0 and Essential 10.

### Mouse primary microglial cultures

Primary microglial cultures were prepared from postnatal day 1 (P1) C57BL/6 mouse pups, as we previously described [21]. Briefly, cortical tissues were dissected in ice-cold Hank’s Balanced Salt Solution (HBSS, Corning). With meninges being carefully removed, the tissues were minced into small pieces and washed three times with cold HBSS. The tissues were then digested with 0.25% trypsin at 37 °C for 25 min with gentle tapping. Enzymatic digestion was terminated by adding an equal volume of complete MEM (Minimum Essential Medium supplemented with 10% fetal bovine serum, 100 U/mL penicillin, and 100 μg/mL streptomycin) containing 0.02% deoxyribonuclease I (DNase I). Cells were pelleted by centrifugation, resuspended in fresh complete MEM, filtered through 70-μm mesh, and seeded into T-75 flasks coated with 0.001% poly-*L*-ornithine (Sigma-Aldrich). After 24 h, the medium was replaced to remove non-adherent cells. On days 4–5, the medium was replaced with fresh complete MEM supplemented with 2 ng/mL granulocyte-macrophage colony-stimulating factor (GM-CSF, R&D Systems). After another 4–5 days of incubation, microglial cells were detached from the underlying astrocytic monolayer by gentle shaking at room temperature, collected, and seeded into 12-well plates at a density of 3 × 10^5^ cells/well in complete MEM containing 0.2 ng/mL GM-CSF. To evaluate the anti-inflammatory effects of 12/15-LOX inhibition, primary microglial cells were pre-treated with ML351 at 10 μM, a concentration at which the compound showed significant neuroprotection against glutamate-induced neurotoxicity [22]. Fifteen minutes later, LPS (100 ng/mL) derived from *Escherichia coli* O111:B4 (Sigma-Aldrich) was added to activate the microglia overnight.

### Quantitative PCR

The mRNA expression levels of examined genes were quantified by quantitative PCR (qPCR) as we described previously [23, 24]. The total RNA from mouse brain tissues was isolated using TRIzol and the PureLink RNA Mini Kit (Invitrogen). RNA purity and concentration were measured by a microvolume spectrophotometer (NanoDrop One, Thermo Fisher Scientific). The complementary DNA (cDNA) was synthesized using the SuperScript III First-Strand Synthesis SuperMix (Invitrogen). The qPCR was performed using cDNA, primers, and 2× SYBR Green SuperMix with a final reaction volume of 10 μL in a CFX96 Touch Real-Time PCR Detection System (Bio-Rad Laboratories). Cycling conditions were set as: 95 °C for 2 min followed by 40 cycles of 95 °C for 15 s and then 60 °C for 1 min. Melting curve analysis was utilized to validate the specificity of the PCR products. Fluorescent data were obtained at the 60 °C step. The cycle of quantification for GAPDH was subtracted from the cycle of quantification measured for each gene of interest to yield ΔCq. The sequences of primers used for qPCR are listed in Table 1.

### ELISA

The protein levels of inflammatory cytokines including interleukin 1β (IL-1β), IL-6, and tumor necrosis factor α (TNF-α) released by cultured microglia into the culture medium were quantified using ELISA kits (R&D Systems, IL-1β: Cat. # SMLB00C; IL-6: Cat. # M6000B-1; TNF-α: Cat. # MTA00B-1), according to the manufacturer’s protocols [25].

### LPS-induced neuroinflammation

LPS (O111:B4, Sigma-Aldrich) was dissolved in sterile isotonic saline and injected into mice intraperitoneally (3 mg/kg). Thirty minutes later, animals were intraperitoneally administered equivalent volume of either vehicle (25% DMSO, 40% PEG-400, 10% Tween-80, 25% ddH_2_O) or ML351 at 50 mg/kg, an effective dose at which the 12/15-LOX inhibitor substantially reduced infarction in a mouse model of permanent focal ischemia [22]. Animals were deeply anesthetized with isoflurane and were perfused with ice-cold PBS 3 h or 6 h after LPS administration. Following transcardiac perfusion, hippocampal tissues were collected, snap-frozen in liquid nitrogen, and stored at −80 °C until RNA extraction [26].

### Pilocarpine-induced status epilepticus

A total of 50 adult C57BL/6 mice (male, ~8 weeks) were used to evaluate the short-term effects of ML351 at one day after SE. Among these, 10 control mice received saline and were randomly divided into two groups (*n* = 5 each) for treatment with vehicle (25% DMSO, 40% PEG-400, 10% Tween-80, 25% ddH_2_O) or ML351 (50 mg/kg, i.p.). The remaining 40 mice were pre-treated with methylscopolamine and terbutaline (2 mg/kg each in 0.9% saline, i.p.) to minimize the peripheral effects of pilocarpine. Thirty minutes later, freshly prepared pilocarpine was administered (280 mg/kg in 0.9% saline, i.p.) to induce seizures in mice. Seizures were observed and classified using a modified Racine scale as described in Table 2 [27]. SE was defined as nonintermittent seizure activities and usually indicated by continual generalized clonic seizures without returning to low-stage seizures. SE proceeded for 1 h and was terminated by diazepam (10 mg/kg, i.p.). Out of 40 pilocarpine-treated mice, 16 died during SE and two did not enter SE. Two hours after SE onset, that is, 1 h after administration of diazepam, the surviving twenty-two SE mice were randomized for treatment with either vehicle (*n* = 11) or compound ML351 (*n* = 11). During recovery from SE, mice were fed moistened rodent chow and given 5% dextrose in lactated Ringer’s solution (Baxter) when needed. Thereafter, another two mice from the Pilocarpine + Vehicle group and one mouse from the Pilocarpine + ML351 died. Twenty-four hours after SE, all the mice were euthanized under deep anesthesia with isoflurane and perfused with ice-cold PBS to wash blood out of the brain. The hippocampal and cortical tissues were then collected for biochemical and histological analyses.

### Tissue processing

Following transcardial perfusion in mice with ice-cold PBS, the right hemisphere of each brain was collected for immunohistochemistry and Fluoro-Jade B staining. Brain tissues were post-fixed in 4% paraformaldehyde (PFA) for 24 h at 4 °C. After fixation, samples were transferred to a 30% sucrose solution in 1× PBS for cryoprotection and stored until further processing. Tissue blocks were embedded in optimal cutting temperature (OCT) compound and were rapidly frozen on dry ice. Coronal brain sections were cut at a thickness of ~ 25 μm using an HM525 NX Cryostat (Thermo Scientific) and stored at −20 °C until staining procedures were performed. Due to an unexpected loss of tissue blocks prior to histological processing (two from the Pilocarpine + Vehicle group and two from the Pilocarpine + ML351 group), the final sample sizes for histological analysis were reduced accordingly.

### Immunohistochemistry

Mouse brain coronal sections were incubated with a blocking solution consisting of 3% BSA and 0.1% Triton X-100 in PBS at room temperature for 60 min. The sections were then incubated in rabbit anti-Iba1 polyclonal antibody (Wako Chemicals, Cat. # 019-19741, 1:500) and GFAP mouse monoclonal antibody (Thermo Fisher Scientific, Cat. # MA5-12023, 1:1000) at 4 °C overnight. Sections were then washed and incubated with goat anti-rabbit Alexa Fluor 488 (Invitrogen, Cat. # A-11034, 1:500) and goat anti-mouse Alexa Fluor 546 (Invitrogen, Cat. # A-11003, 1:500) for 1 h at room temperature. After washing for 3 times, sections were stained with DAPI (1 μg/mL in PBS, Invitrogen) for 10 min and carefully mounted onto slides using ProLong Gold antifade mountant (Invitrogen, Cat. # P36930). Digital images were captured using a fluorescence microscope (BZ-X710, Keyence), and the image processing and quantitative analyses were performed using the ImageJ/Fiji software (Version 1.54 f).

### Fluoro-Jade B staining

Fluoro-Jade B (FJB) reagent is a neuron-specific anion which can selectively label degenerating neurons and was thus used to detect SE-induced neuronal death in this study as we previously described [28]. In brief, coronal brain sections were successively immersed in 80% alcohol containing 1% NaOH for 5 min, in 70% alcohol for 2 min, and in distilled water for 2 min. Sections were then incubated in 0.06% potassium permanganate for 30 min with gentle agitation. After rinsing in distilled water for 1 min, sections were transferred to the FJB solution (0.0004%, *w/v*, in distilled water with 0.1% acetic acid) for 30 min with gentle agitation in the dark. Sections were rinsed with three changes (1 min each) of distilled water, rapidly dried, and covered by coverslip with DPX mountant. Images were captured by a fluorescence microscope (BZ-X710, Keyence), and the FJB-positive cells were counted in the sections between bregma −1.5 and −3.0.

### Behavioral tests

A total of 60 adult C57BL/6 mice were utilized for the behavioral study. Among them, 10 mice received saline and vehicle as the control animals, and the rest received pilocarpine for SE induction. Among the pilocarpine injected mice, 30 mice survived and were randomized into two groups (*n* = 15 each) to receive either vehicle or ML351 (50 mg/kg, i.p.) for five days. The mice recovered for 28 days and were closely monitored. During this time, 2 mice from Pilocarpine + Vehicle group and 3 mice from Pilocarpine + ML351 group died. The final group size was as follows: Control = 10, Pilocarpine + Vehicle = 13, Pilocarpine + ML351 = 12. A battery of behavioral tests were carried out to assess the neurobehavioral comorbidities in mice beginning 28 days after pilocarpine-induced SE in a soundproof room with a neutral environment as we previously described [29]. As described below, these behavioral tests were performed only once on different days to minimize any carry-over effects [30].

The open field test was first conducted on day 28 to assess chronic general locomotor activity and anxiety-like behavior. In brief, mice were placed individually in the center of a square open field (48 cm× 48 cm× 48 cm) and allowed to explore freely for 5 min. The box was cleaned after completion of experiment for each animal using 70% ethanol. The moving traces of mice were monitored with an infrared camera, and the behavior analyses were conducted using a video tracking system. Total travel distance and time spent in the center zone were recorded. Reduced time in the center zone was interpreted as increased anxiety-like behavior.

The light/dark box test was also used to evaluate anxiety-like behavior. The test was conducted on day 29 after SE to evaluate anxiety-like behavior. The apparatus consisted of two compartments: a brightly lit chamber and a dark chamber, connected by a small opening. Mice were placed in the light compartment and allowed to explore both chambers for 10 min. Time spent in the light compartment was recorded. Reduced time in the light zone was interpreted as increased anxiety-like behavior.

The novel object recognition (NOR) test was performed on days 30–32 using the same apparatus as open field test to assess recognition memory. The test consisted of three phases: habituation (day 30), training (day 31) and testing (day 32). During habituation, mice were allowed to explore an empty arena for 10 min. On the second day, during the training phase, two identical objects were placed in the arena, and mice were allowed to explore for 10 min. On the third day, one familiar object was replaced with a novel object, and mice were allowed to explore for 10 min. The recognition index was calculated as: recognition index (%) = (Time spent with novel object) / (Time spent with both objects) × 100.

To assess short-term spatial working memory, mice were placed in a Y-shaped maze with three identical arms (each about 30 cm long, 6 cm wide, and 6 cm high) on day 33 post SE. Mice were allowed to explore the maze freely for 8 min. The sequence and number of arm entries were recorded. Spontaneous alternation was defined as consecutive entries into all three arms without repetition. The percentage of spontaneous alternation was calculated as: spontaneous alternation (%) = (Number of alternations) / (Total arm entries - 2) × 100.

### Nissl staining

After completion of all behavioral tests, i.e., 34 days after SE, brain tissues from the same mice used for behavioral experiments were harvested for Nissl staining. Eight samples per group were taken and sectioned coronally at ~ 25 μm thickness. Free-floating sections were washed three times in PBS, mounted onto gelatin-coated slides, and air-dried overnight at room temperature. The following day, slides were rinsed with tap water and dehydrated in 100% ethanol (3 × 3 min), followed by clearing in xylene (3 × 3 min). Sections were then rehydrated through 100% ethanol (3 × 3 min) and 95% ethanol (3 min) and rinsed again with tap water. Staining was performed with 0.1% cresyl violet solution for 1 h at 37 °C. After a brief rinse in water, sections were differentiated in 70% ethanol (5 s) and 95% ethanol (5 s), dehydrated in 100% ethanol (3 × 2 min), cleared in xylene (3 × 1 min), and cover slipped using a mounting medium. Images were taken using a fluorescence microscope (BZ-X710, Keyence). The captured photos were converted to 8-bit, and the contrast was adjusted to identify the cells using ImageJ/Fiji software (Version 1.54 f). Cells with well-defined cell bodies, darkly stained Nissl substance and the presence of nucleus and nucleolus were counted as surviving neurons. On the contrary, cells featured with shrunken cell bodies, pale or dispersed Nissl substance (chromatolysis), condensed nuclei (pyknosis), and vacuolization were considered as degenerated neurons.

### Statistical analysis

All analyses were performed using GraphPad Prism (Version 10.6.1). For comparisons between two groups, an unpaired two-tailed Student’s *t*-test was used. When comparing more than two groups, one-way ANOVA followed by Tukey’s or Dunnett’s *post hoc* test was applied. For experiments involving two independent variables, two-way ANOVA with Šídák’s multiple comparison test was performed. Outliers were identified and removed using the ROUT method. Statistical significance was set at *P*< 0.05. All data are presented as mean ± standard deviation (SD).

## RESULTS

### 12/15-LOX in microglia contributes to inflammatory cytokine production

**Fig. 1:**
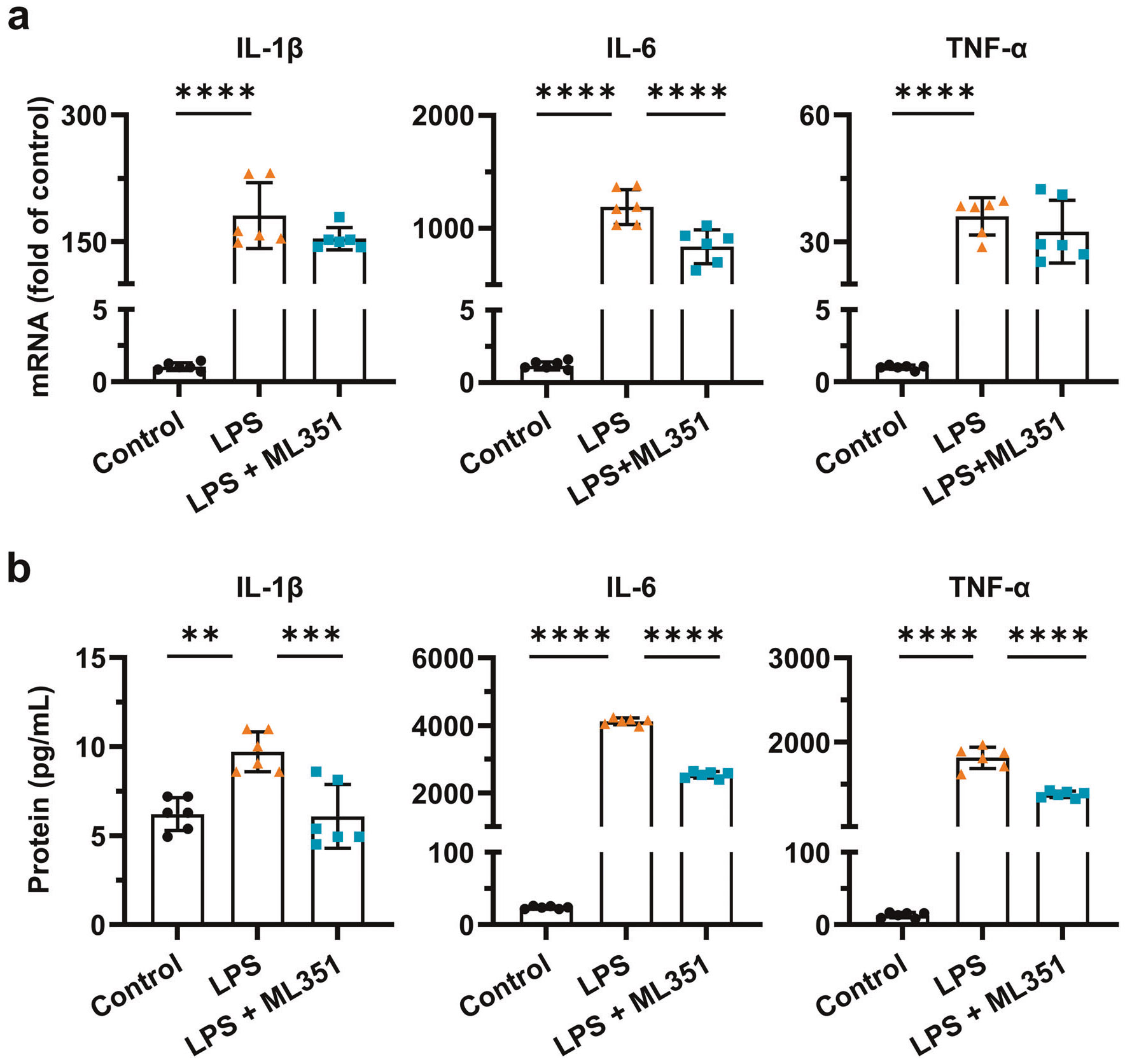
Inhibition of 12/15-LOX attenuates inflammatory cytokines produced by activated microglia. Mouse primary microglial cultures were pre-treated with compound ML351 (10 μM) for 15 min, followed by coincubation with LPS (100 ng/mL) overnight. **a** The mRNA expression levels of IL-1β, IL-6, and TNF-α were measured using qPCR. **b** The protein levels of IL-1β, IL-6, and TNF-α in the culture medium were quantified by ELISA. Data are presented as mean ± SD (*n* = 6). Statistical analyses were performed using one-way ANOVA followed by Tukey’s *post hoc* test (**P < 0.01; ***P < 0.001; ****P < 0.0001).

Microglia represent the first line of the immune defense system in the CNS and can also be involved in the pathophysiological processes. Their initial activation is often considered as a self-protection mechanism against acute neurological insults. However, prolonged and excessive microglial activities might orchestrate to sustain neuroinflammation by releasing pro-inflammatory cytokines like IL-1β, IL-6, and TNF-α, exacerbating the underlying conditions [31]. 12/15-LOX activation is known for its essential role in microglia-mediated oxidative stress and inflammatory processes [32]. To investigate the potential of microglial 12/15-LOX as a new anti-inflammatory target, we first treated primary microglia from neonatal mouse brains with ML351 followed by overnight LPS stimulation to induce inflammatory phenotype. LPS stimulation substantially increased the expression of cytokines IL-1β, IL-6, and TNF-α at both the mRNA (Fig. 1a) and protein levels (Fig. 1b). Treatment with ML351 resulted in a significant reduction in mRNA expression of IL-6 (*P* < 0.0001) as well as a downward trend of IL-1β (*P* = 0.1491) and TNF-α (*P* = 0.4466) mRNA levels in LPS-stimulated microglia (Fig. 1a). Interestingly, ELISA analyses revealed that ML351 significantly reduced the protein levels of all three cytokines (IL-1β, *P* = 0.0008; IL-6, *P* < 0.0001; TNF-α, *P* < 0.0001) secreted in the culture medium by LPS-activated microglia (Fig. 1b). The discrepancy between mRNA and protein levels of these cytokines may be attributed to post-transcriptional regulatory mechanisms that can modulate protein abundance independently of mRNA expression levels [33]. Nonetheless, these results together demonstrate that 12/15-LOX inhibition by compound ML351 is sufficient to suppress the inflammatory response among activated microglia.

Inhibition of 12/15-LOX reduces LPS-triggered neuroinflammation The convincing anti-inflammatory effects of compound ML351 on microglia-derived cytokines in vitro encouraged us to continue investigating the neuroinflammatory role of 12/15-LOX in vivo. We chose to study the LPS-induced neuroinflammation in mice because this model precisely mimics the neuroinflammatory environment observed in many neurological disorders [34]. We first examined the time-course expression of 12/15-LOX (encoded by Alox15) and several common inflammatory mediators within the brain after LPS exposure in mice, because the results might help us to determine the possible time window for anti-inflammatory treatment as we previously practiced [35]. As such, we measured the mRNA levels of these inflammatory genes along with *Alox15* in the hippocampus at different time points following LPS administration in mice. We initially focused our analyses on hippocampal region because neuroinflammatory conditions often greatly alter transcriptional profile of inflammatory genes in the hippocampus, thereby affecting neurogenesis and ultimately brain functions [36].

**Fig. 2:**
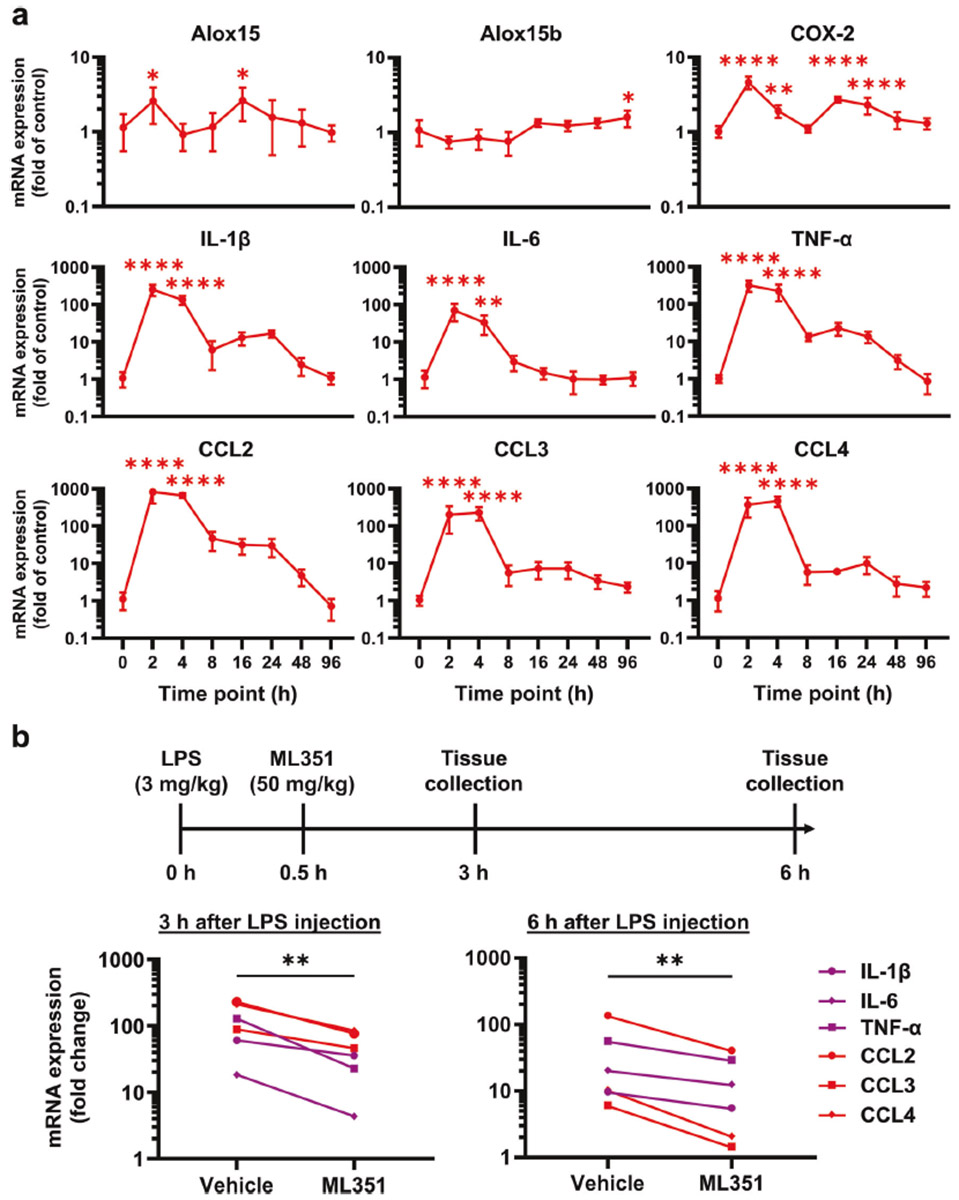
The 12/15-LOX enzyme regulates expression of pro-inflammatory genes in the brain. **a** Adult C57BL/6 mice were systemically treated with LPS (3 mg/kg, i.p.), and the mRNA levels of Alox15, Alox15b, COX-2, cytokines (IL-1β, IL-6, TNF-α), and chemokines (CCL2, CCL3, CCL4) were measured using qPCR at eight different time points (0, 2, 4, 8, 16, 24, 48, 96 h) after LPS administration. All the data were presented as mean ± SD (*n* = 6). Statistical analyses were performed by one-way ANOVA followed by *post hoc* Dunnett’s test (*P < 0.05; **P < 0.01; ****P < 0.0001). **b** LPS (3 mg/kg, i.p.) was administered to mice for induction of neuroinflammation. Thirty minutes later, a single dose of ML351 (50 mg/kg, i.p.) was given. Hippocampal tissues were collected at two different time points: 3 h and 6 h after LPS administration. The mRNA levels of cytokines (IL-1β, IL-6, and TNF-α) and chemokines (CCL2, CCL3, and CCL4) were quantified using qPCR and indicated as fold mRNA induction, which was calculated as the mean mRNA level in LPS mice compared with that in control mice without LPS (*n* = 8 and 6 for 3 h and 6 h, respectively). Statistical significance was determined by ratio paired *t* test (**P < 0.01).

We first found a 2.5-fold induction of *Alox15* mRNA at 2 h after LPS injection (Fig. 2a). Interestingly, the upregulation of *Alox15* coincided with the peak expression of other key pro-inflammatory mediators, such as cyclooxygenase-2 (COX-2), cytokines (IL-1β, IL-6, TNF-α), and chemokines (CCL2, CCL3, CCL4) (Fig. 2a). Intriguingly, there was a second induction peak of *Alox15* at 16 h after LPS injection when most inflammatory mediators remained elevated, except for IL-6 (Fig. 2a). In contrast, *Alox15b*, which encodes an enzyme structurally homologous to human 15-LOX-2 but with distinct regiospecificity, remained at basal expression levels throughout the time course, suggesting that the inducible *Alox15* isoform is specifically associated with inflammation and its resolution. The early induction of *Alox15* along with key pro-inflammatory genes at 2 h after inflammatory stimulation in this model suggests its involvement in the initiation of the neuroinflammatory response and highlights a critical window for therapeutic intervention targeting 12/15-LOX during the peak of inflammatory reactions.

**Fig. 3:**
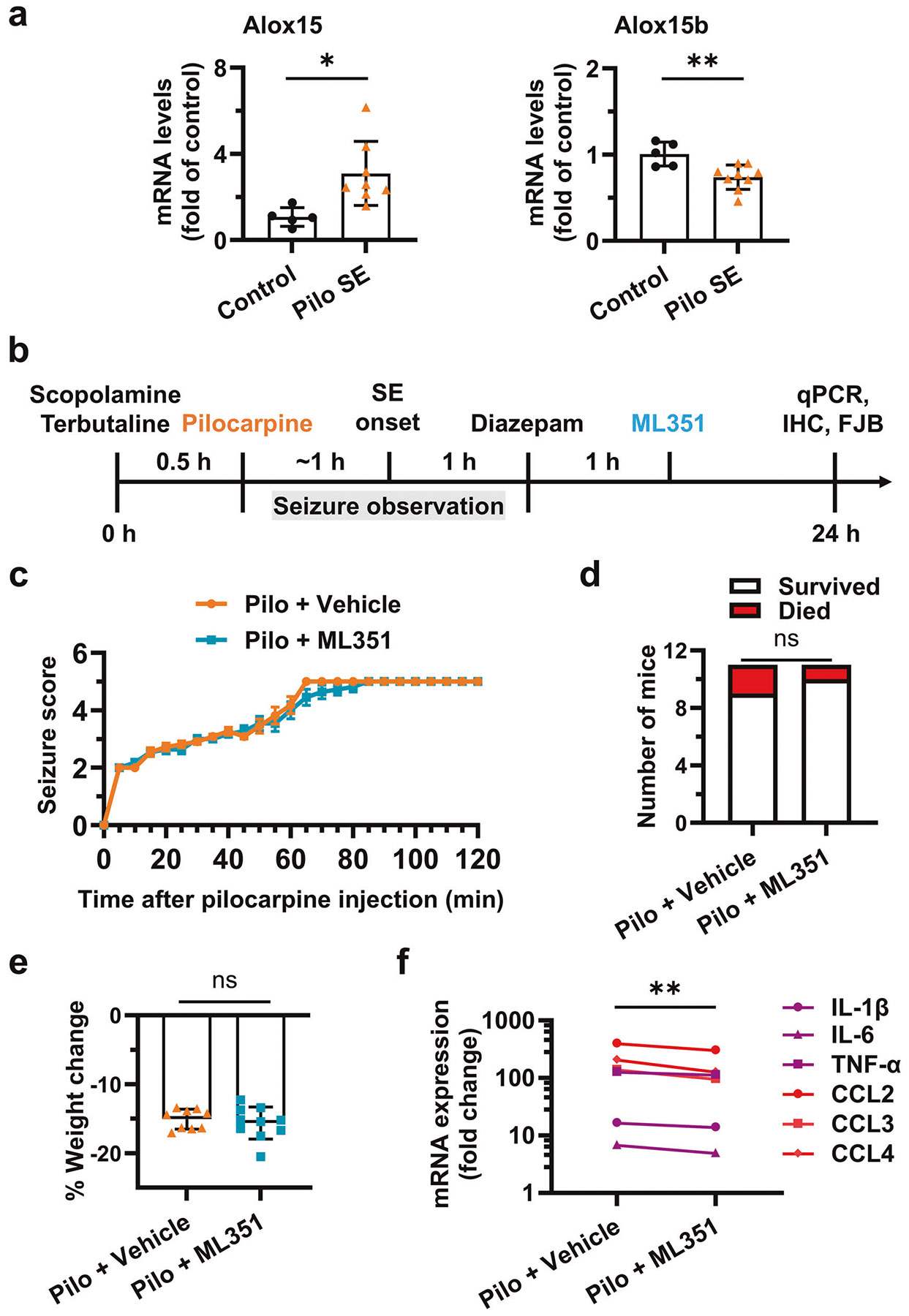
Inhibition of 12/15-LOX reduces seizure-induced neuroinflammation. **a** The mRNA expression of Alox15 and Alox15b in the hippocampus was measured 1 day after pilocarpine-induced SE in mice by qPCR. Data are presented as mean ± SD (*n* = 5 and 9 for control and SE, respectively). Statistical significance was determined using unpaired *t* test (*P < 0.05; **P < 0.01). **b** Mice were treated with methylscopolamine and terbutaline (2 mg/kg each, i.p.) and 30 min later by pilocarpine (280 mg/kg, i.p.) for SE induction. SE proceeded for 60 min and was interrupted by diazepam (10 mg/kg, i.p.). After recovery for 1 h, mice were randomly treated with vehicle or compound ML351 (50 mg/kg, i.p.). All mice were sacrificed 24 h after SE, and brain tissues were collected for neuropathological analyses. **c** Mice were monitored for behavioral seizures after pilocarpine injection, and seizure scores were tabulated every 5 min prior to treatment. Data are plotted as mean ± SD (*n* = 11). **d** Post-treatment mortality was recorded 24 h after SE. In the Pilocarpine + Vehicle group, 2 out of 11 mice (18.2%) died, whereas in the Pilocarpine + ML351 group, 1 out of 11 mice (9.1%) died. Fisher’s exact test was performed (*P* > 0.9999; ns = not significant). **e** Mice were weighed immediately before seizure induction and 24 h after SE. Percentage weight loss during this period was calculated and compared using unpaired *t* test (*P* = 0.5438; ns = not significant). Data are presented as mean ± SD (*n* = 9 and 10). **f** Cytokines (IL-1β, IL-6, and TNF-α) and chemokines (CCL2, CCL3, and CCL4) in the hippocampus were measured by qPCR for their mRNA expression 24 h after pilocarpine-induced SE in mice. Statistical significance was determined by ratio paired *t* test (**P < 0.01).

Based on the time-course analysis data (Fig. 2a) and pharmacokinetic parameters of ML351, which has a plasma half-life of 1.1 h and a brain/plasma ratio of 2.8 in mice [22], we decided to treat the animals with ML351 (50 mg/kg) 30 min after LPS injection. Hippocampal tissues were then collected 3 h after LPS administration, i.e., 1 h after the peak of inflammatory induction. The qPCR analysis revealed that treatment with compound ML351 largely reduced (*P* = 0.0024) the overall mRNA expression of key pro-inflammatory cytokines and chemokines in the hippocampus (Fig. 2b). We next wanted to know whether a single dose of ML351 can have any longer effect on these inflammatory genes. To answer this question, we collected hippocampal tissues from another set of mice 6 h after LPS administration. Interestingly, treatment with ML351 significantly suppressed (*P* = 0.0028) the overall expression of these inflammatory genes even 6 h after LPS injection (Fig. 2b), indicating that early treatment with the 12/15-LOX inhibitor has a long-lasting effect downregulating the inflammatory gene expression. Together, these findings support the feasibility of therapeutically targeting 12/15-LOX in an animal model of neuroinflammation and validate the translational relevance of our in vitro observations. Post-SE inhibition of 12/15-LOX reduces brain cytokine surge Other than just a key hallmark of the epileptic brain, neuroinflammation is also known as an essential contributor to the neuropathogenesis of prolonged seizures and the long-term consequences, including neuronal death, disrupted neural networks, and behavioral impairments [37, 38]. We next sought to evaluate the therapeutic potential of 12/15-LOX inhibition in a model of SE, as prolonged seizures are known to trigger robust neuroinflammatory responses and neuronal damage, particularly within the hippocampus. SE was induced by pilocarpine injection and allowed to proceed for 1 h before being terminated with diazepam. Using this protocol, we found that the *Alox15* mRNA expression in the hippocampus was upregulated (*P* = 0.0137) nearly 3 times by SE, measured 1 d after SE onset by qPCR. Surprisingly, in the meantime, the expression of *Alox15b* was modestly decreased (*P* = 0.0051) in the hippocampus (Fig. 3a). These results suggest a conceivable role for the inducible 12/15-LOX but not 15-LOX-2 isozyme in the early inflammatory response following prolonged SE.

To assess the anti-inflammatory efficacy of compound ML351 in this model, mice received a single dose of ML351 (50 mg/kg, i.p.) or vehicle 1 h after SE was terminated by diazepam, i.e., 2 h after the SE onset (Fig. 3b). Behavioral seizure monitoring confirmed no significant difference in seizure progression or severity between groups prior to treatment (Fig. 3c), validating randomization. As anticipated, pilocarpine-induced SE caused substantial post-SE mortality (~18.2%) and weight loss (~15.1%) within 24 h in vehicle-treated mice, which were not significantly changed by treatment with ML351 (Fig. 3d, e). All the surviving animals were sacrificed 24 h after SE, and the brain tissues were harvested for further biochemical and histological analyses. The qPCR analysis of hippocampal tissues from SE mice revealed a consistent upregulation of inflammation-associated genes including cytokines (IL-1β, IL-6, TNF-α) and chemokines (CCL2, CCL3, CCL4). However, the overall induction of these key inflammatory mediators was largely abolished (*P* = 0.0033) by the treatment with compound ML351 (Fig. 3f), suggesting an essential role of 12/15-LOX in seizure-provoked brain inflammation. Given the pivotal roles of these cytokines and chemokines in seizure generation and recurrence [39], targeting 12/15-LOX after SE might provide a new antiseizure, potentially, antiepileptogenic strategy.

### 12/15-LOX inhibition blunts reactive gliosis after SE

**Fig. 4:**
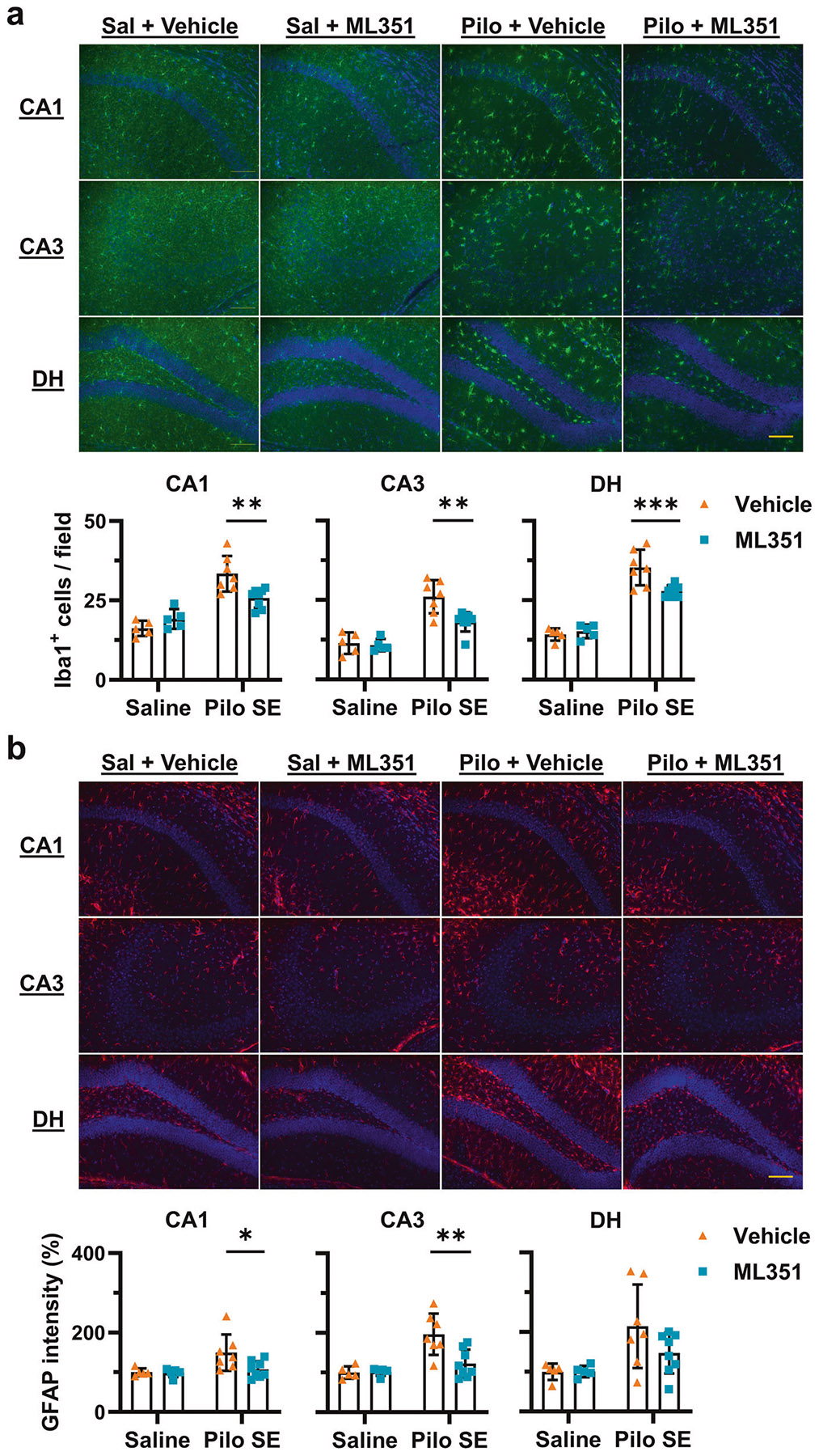
Blockade of 12/15-LOX eases SE-triggered reactive gliosis. Immunostaining for Iba1 and GFAP was performed 1 day after SE to visualize reactive microglia and astrocytes, respectively. **a** Representative images of microglia (Iba1^+^) as highlighted by green fluorescence across hippocampal subregions CA1, CA3, and dentate hilus (DH). Iba1^+^ cells were counted in the selected areas for indication of reactive microgliosis after SE. **b** Representative images showing activation of astrocytes (GFAP^+^) in the hippocampus, illustrated by red fluorescence. The overall intensity of fluorescence was used to indicate the severity levels of astrogliosis. Note that nuclear counterstaining was performed with DAPI (blue fluorescence). Imaging parameters remained same across samples. Scale bar = 100 μm. All data were presented as mean ± SD (*n* = 5, 5, 7, 8). Statistical significance was determined using two-way ANOVA followed by Šídák’s multiple comparison test (*P < 0.05; **P < 0.01; ***P < 0.001).

Reactive gliosis is characterized by morphological and functional changes in glial cells, particularly microglia and astrocytes, in response to acute brain insults including seizures. Activation of these glial cells is widely considered as a key hallmark of epileptogenesis and might contribute to the seizure recurrence [40]. Considering gliosis upregulates several proteins including ionized calcium-binding adaptor molecule 1 (Iba1) in microglia and glial fibrillary acidic protein (GFAP) in astrocytes, we next evaluated the effects of 12/15-LOX inhibition on these two biomarkers of gliosis after SE using immunofluorescence staining. We found that SE induced robust microgliosis in the hippocampal tissues from vehicle-treated mice, evidenced by morphological transformation of microglia from ramified state to activated amoeboid state (Fig. 4a). Two-way ANOVA revealed a significant main effect of SE and a significant interaction between SE and ML351 treatment in all examined hippocampal regions [CA1: *F*_(1,21)_ = 11.39, *P* = 0.0029; CA3: *F*_(1,21)_ = 5.761, *P* = 0.0257; DH: *F*_(1,21)_ = 9.046, *P* = 0.0067]. *Post hoc* Šídák’s multiple comparisons confirmed that ML351 significantly reduced SE-induced microglial activation by ~45% in CA1 (*P* = 0.0019), ~54% in CA3 (*P* = 0.0010), and ~35% in DH (*P* = 0.0008) (Fig. 4a). Similarly, SE markedly increased GFAP immunoreactivity in all subregions, with two-way ANOVA revealing a significant main effect of SE (Fig. 4b). A significant interaction between SE and ML351 was observed in CA3 [*F*_(1,21)_ = 6.896, *P* = 0.0158], with a trend toward interaction in CA1 (*P* = 0.0888) but not in DH (*P* = 0.2090). Šídák’s multiple comparisons test demonstrated that ML351 treatment significantly blunted SE-induced astrogliosis, reducing GFAP intensity by ~85% in CA1 (*P* = 0.0165) and ~76% in CA3 (*P* = 0.0012), whereas the reduction in DH did not reach significance (*P* = 0.1102) (Fig. 4b). The substantial inhibitory effects of ML351 on both glial biomarkers suggest that 12/15-LOX-mediated pathways might play an essential role in reactive gliosis following prolonged seizures.

### Inhibition of 12/15-LOX after SE confers neuroprotection

**Fig. 5:**
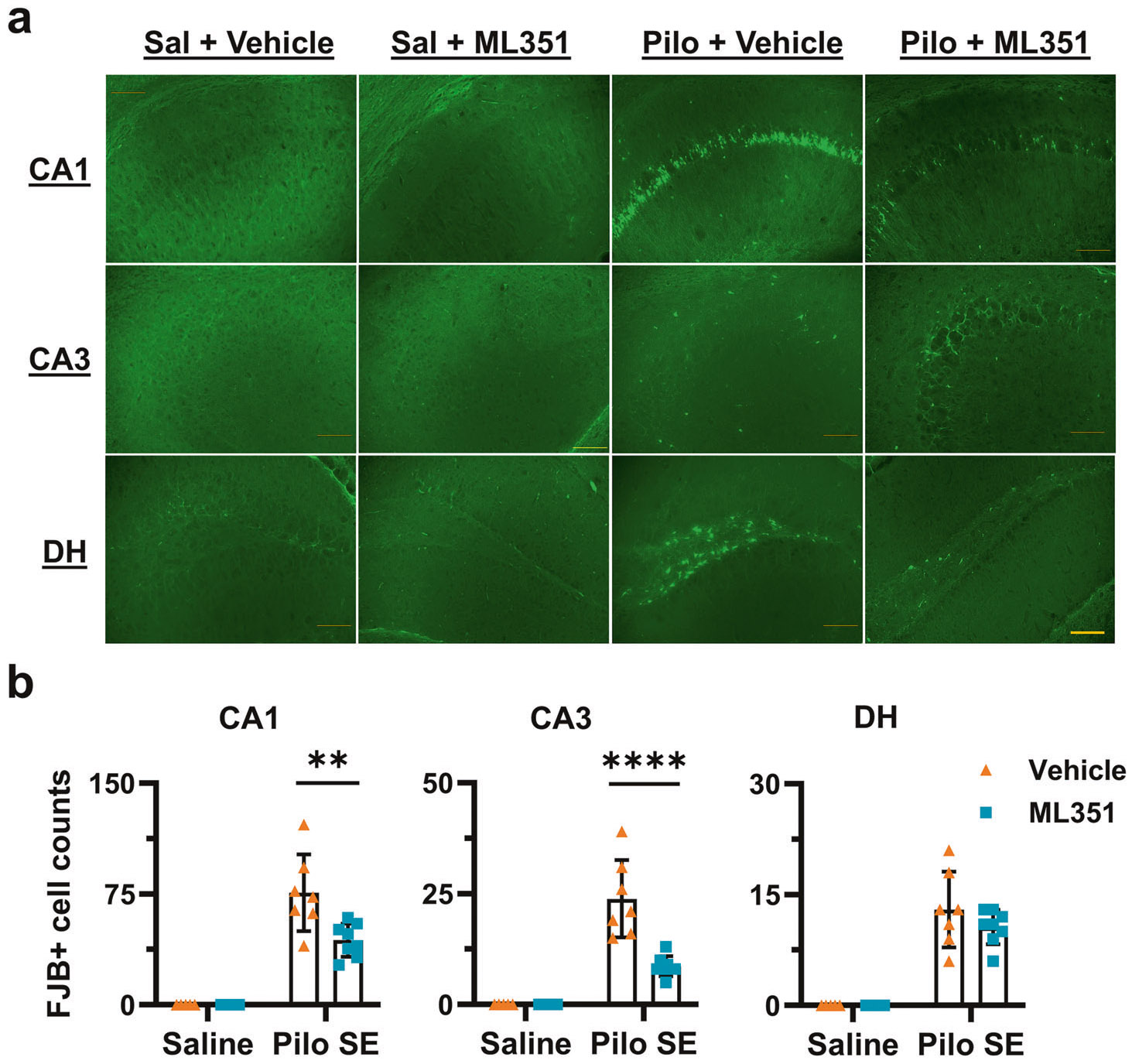
Post-SE inhibition of 12/15-LOX is neuroprotective. **a** Representative images showing degenerating neurons across the hippocampus, detected by Fluoro-Jade B (FJB) staining 1 day after pilocarpine-induced SE in mice. Scale bar = 100 μm. **b** FJB-positive cells per section (bregma between – 1.5 and – 3) were counted in the hippocampal subregions CA1, CA3, and DH for comparisons. All data were presented as mean ± SD (*n* = 5, 5, 7, 8). Statistical significance was determined using two-way ANOVA followed by Šídák’s multiple comparison test (**P < 0.01; ****P < 0.0001).

Severe and repetitive seizures are known to initiate a cascade of molecular, cellular, and histological alterations that can culminate in neuronal death. Neuronal loss in the hippocampus has been implicated in epileptogenesis (i.e., the development of chronic epilepsy) and impairment of cognitive functions [41]. To determine whether 12/15-LOX inhibition could mitigate seizure-induced neuronal injury, we assessed acute hippocampal neurodegeneration 24 h after pilocarpine-induced SE by tissue staining with Fluoro-Jade B (FJB), a fluorescent dye that selectively labels degenerating neurons [42]. Positively stained cells were counted in each area to determine the overall neurodegeneration level, and we found that vehicle-treated SE mice exhibited extensive neuronal degeneration across CA1, CA3, and DH (Fig. 5a). ML351 treatment significantly reduced FJB-positive cells by ~38% in CA1 (*P* = 0.0012) and ~64% in CA3 (*P* < 0.0001), while a~22% reduction was observed in DH but did not reach significance (*P* = 0.2738) (Fig. 5b). A two-way ANOVA analysis presents a significant interaction between SE and ML351 in CA1 [F (1,21) = 6.553; *P* = 0.0182] and CA3 [F (1, 21) = 14.81; *P* = 0.0009], demonstrating that the neuroprotective effect of ML351 in those regions occurred specifically in the context of SE. These findings suggest that post-SE administration of compound ML351 confers considerable protection of hippocampal cells from prolonged seizure-triggered acute cell death.

### 12/15-LOX inhibition following SE is anxiolytic

**Fig. 6:**
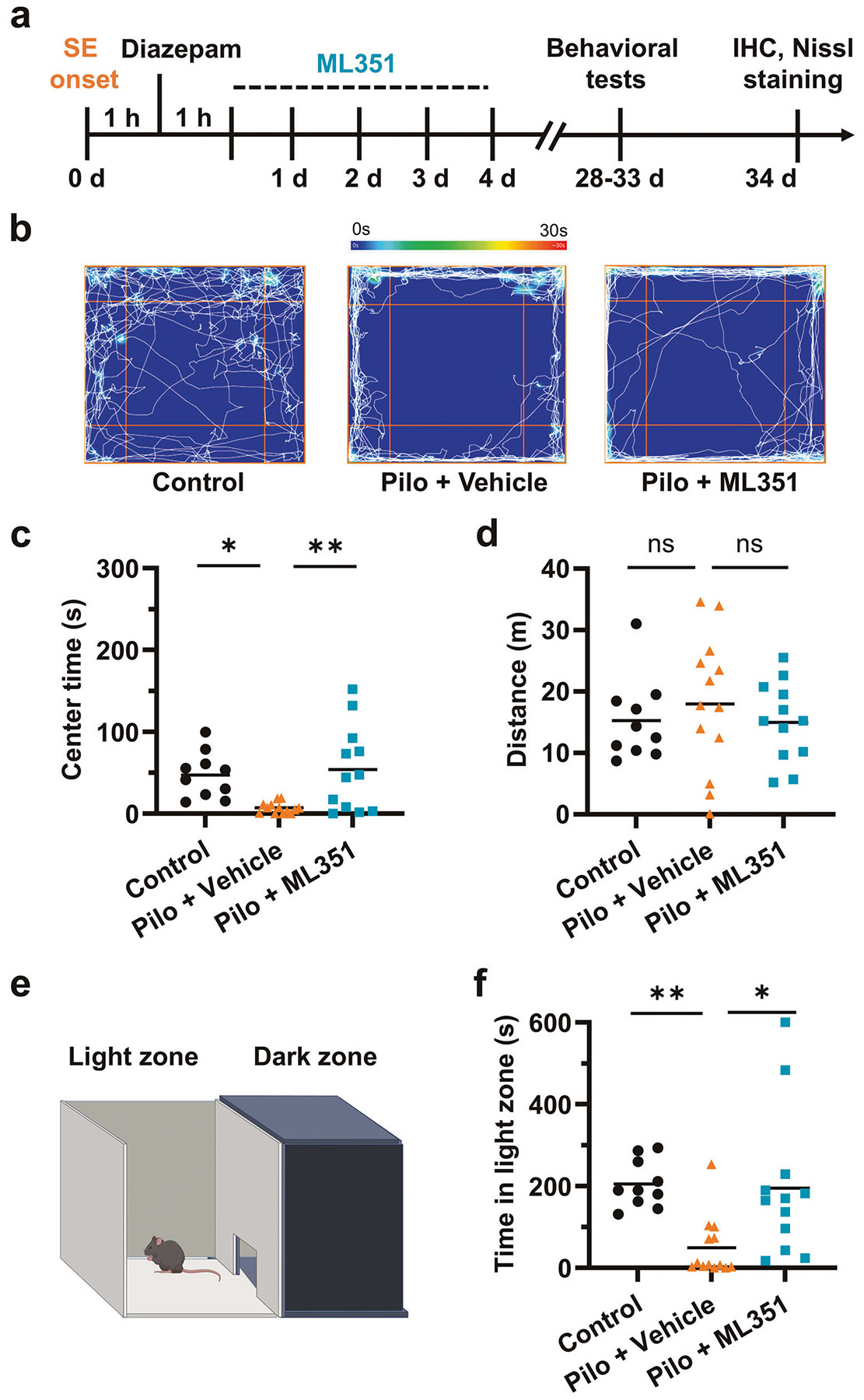
Inhibition of 12/15-LOX after SE diminishes anxiety-like behavior. **a** Diagram showing the timeline for post-SE treatment and behavioral tests. After recovery for 1 h from an episode of SE (1 h), mice were randomly treated with compound ML351 (50 mg/kg, i.p.) for 5 days. **b** Open-field test (OFT) was performed for 5 min to assess the effects of 12/15-LOX inhibitor ML351 on day 28 after SE. Representative images showing track plots with heatmap during the OFT. **c** Time spent in the center of the open field was analyzed as an indicator for anxiety. **d** Total distance travelled during the OFT. **e** Light/dark box test was performed for 10 min on day 29 after SE. **f** Reduced total time spent in the light compartment was considered as another sign for anxiety-like behavior. All data were shown in dot plots with means indicated (*n* = 10, 13, 12). Statistical significance was determined using one-way ANOVA followed by Tukey’s *post hoc* test (*P < 0.05; **P < 0.01; ns = not significant).

Behavioral disturbances, such as anxiety, depression, and cognitive deficits, are among the most debilitating comorbidities of epilepsy and greatly impact the quality of life of patients who also have to deal with seizures [43]. To determine whether 12/15-LOX plays a role in the development of these behavioral abnormalities, we conducted a battery of behavioral tests 4 weeks after pilocarpine-induced SE (Fig. 6a). Among these, the open-field test was first performed to assess the effects of ML351 on the spontaneous locomotor activity and anxiety-like behavior in mice after SE (Fig. 6b). One-way ANOVA confirmed a significant difference among groups [F(2,30) = 5.816, *P* = 0.0073]. Mice that previously experienced SE spent approximately 76% less time in the center zone of the arena compared to the normal control animals (*P* = 0.0185), reflecting heightened anxiety-like symptoms. Interestingly, the SE-triggered anxiety-like behavior was significantly reduced (*P* = 0.0167) among ML351-treated mice, which spent similar total time in the center zone to that of the non-SE animals (Fig. 6c). The total travel distance did not differ between these groups; therefore, the alleviation of anxiety symptoms by treatment with compound ML351 was not due to any difference in general locomotor activity (Fig. 6d).

We then performed the light-dark box test to further investigate anxiety-like behavior among these mice after SE (Fig. 6e), as rodents with higher anxiety status prefer to spend less time in the light compartment [44]. The data were analyzed by one-way ANOVA indicating significant difference among groups [*F*_(2,31)_ = 5.884, *P* = 0.0068]. *Post hoc* Tukey’s test was performed to compare between groups. We found around 74% reduction in time spent in the light compartment among vehicle-treated SE mice when compared to the non-SE cohorts (*P* = 0.0150) (Fig. 6f). However, the SE mice that received ML351 treatment dramatically restored their presence in the lighted compartment (*P* = 0.0175) (Fig. 6g), suggesting a considerable relief of anxiogenic behavior. The results from open field and light-dark box tests together provide evidence supporting an anxiolytic effect of compound ML351 among mice after SE.

### 12/15-LOX inhibition prevents SE-associated cognitive decline

**Fig. 7:**
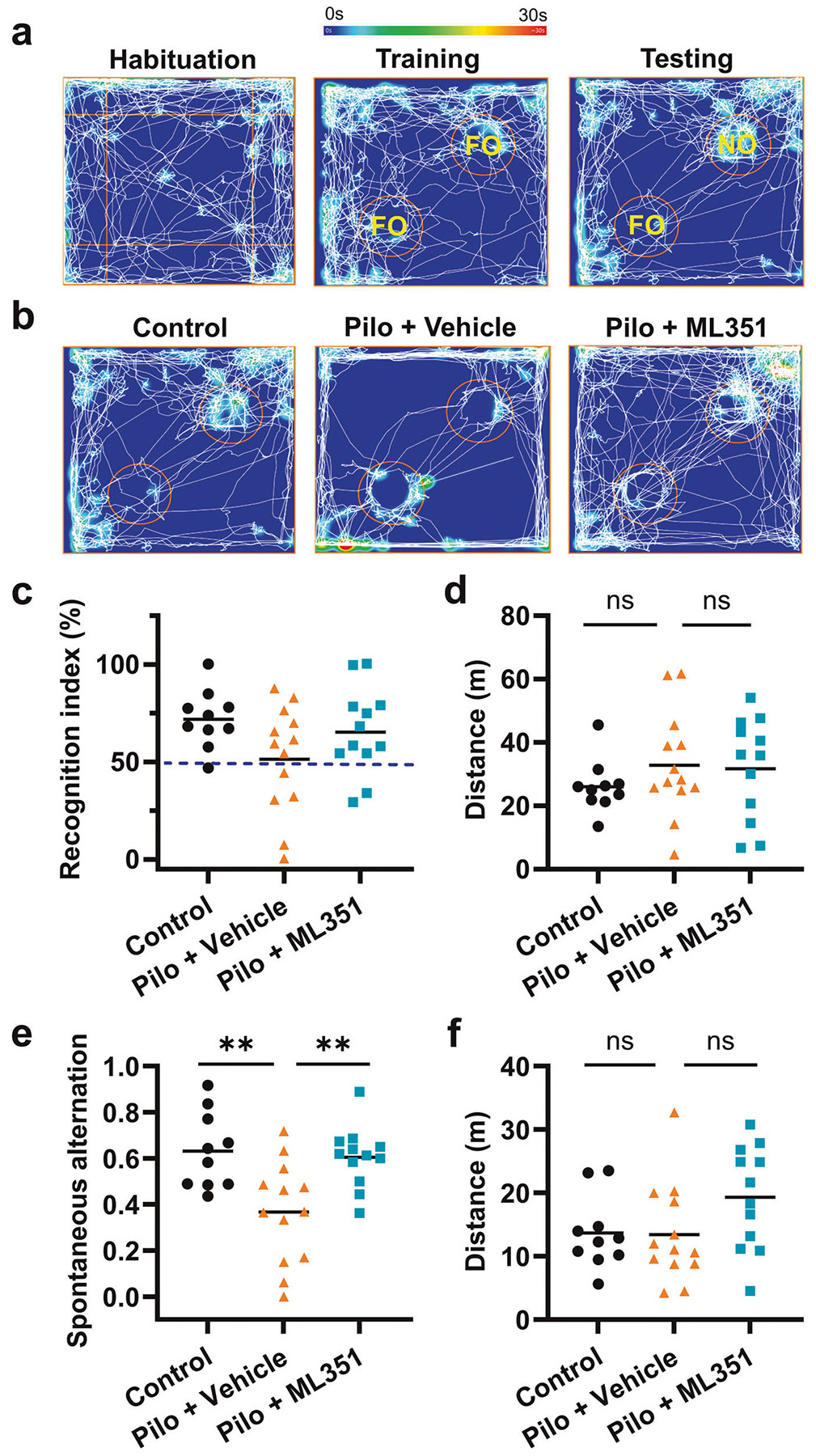
Post-SE treatment with the 12/15-LOX inhibitor restores cognitive functions. **a** Representative images showing the three sessions of the novel object recognition (NOR) test: habituation, training, and testing (10 min each), which was performed 30-32 days after SE. Note that the positions of familiar object (FO) and novel object (NO) are highlighted. **b** Representative images from each experimental group showing track plots with heatmap during the testing session. **c** Recognition index (RI) was calculated for comparisons. **d** Total travel distance during the testing session. **e** Y-maze test was performed for 8 min 33 days after SE. Spontaneous alternation in Y-maze test was calculated to indicate short-term memory. **f** Total travel distance during the Y-maze test. All data were shown in dot plots with means indicated (*n* = 10, 13, 12). Statistical significance was determined using one-way ANOVA followed by Tukey’s *post hoc* test (**P < 0.01; ns = not significant).

We next examined the cognitive function of these mice using novel object recognition (NOR) and Y-maze tests. The NOR is commonly used to assess the learning and memory functions in rodents and is typically conducted in three sessions: habituation, training, and testing (Fig. 7a). During training, animals are allowed to explore two identical objects, whereas in the testing session, one identical object is replaced by a novel object. Rodents naturally show a preference for the novel object, making NOR a reliable way to evaluate their ability to recognize the familiar object (Fig. 7a). Indeed, during testing session, the recognition index of all control mice bar one (9 out of 10) was higher than 50%, and the average recognition index was 72% (Fig. 7b, c), suggesting a preference for the novel object at normal conditions. However, mice after SE had an average recognition index of 51%, with 5 below and 8 above 50% (Fig. 7b, c), indicating a lack of recognition of the familiar object among these animals. Interestingly, treatment with the 12/15-LOX inhibitor in SE mice led to a trend of cognitive improvement, as these animals showed an average recognition index of 65%. Although the difference between the vehicle and ML351 groups did not reach statistically significant (*P* = 0.2849), only 2 out of 12 ML351-treated SE mice had a recognition index below 50% (Fig. 7c), supporting that SE-impaired recognition was partially restored by the inhibitor. The total travel distance during the testing session did not differ between experimental groups (Fig. 7d), supporting that the improvement in NOR test by ML351 treatment was not a direct outcome of any difference in general locomotor activity.

We then investigated the spatial memory function of these animals using a Y-maze, which consists of three arms. The Y-maze test assesses the animal’s ability to remember the arm that it has recently explored and to preferentially visit the previously unexplored arm. This behavior, known as spontaneous alternation, is considered an indicator of short-term spatial memory. In line with findings from the NOR test, mice after SE exhibited a significant reduction (*P* = 0.0040) in spontaneous alternation behavior when compared to the non-SE cohorts (Fig. 7e), indicative of impaired short-term spatial memory caused by prolonged seizures. Consistently, this deficit in spontaneous alternation was also significantly prevented (*P* = 0.0066) by treatment with compound ML351 (Fig. 7e). The total travel distance in the Y-maze test remained similar across experimental groups (Fig. 7f), indicating that the improved memory performance was not due to any difference in the overall motor activity. Together, these findings demonstrate that selective inhibition of 12/15-LOX is adequate to decrease the long-term cognitive decline caused by SE.

### 12/15-LOX inhibition after SE reduces chronic gliosis and neuronal loss

**Fig. 8:**
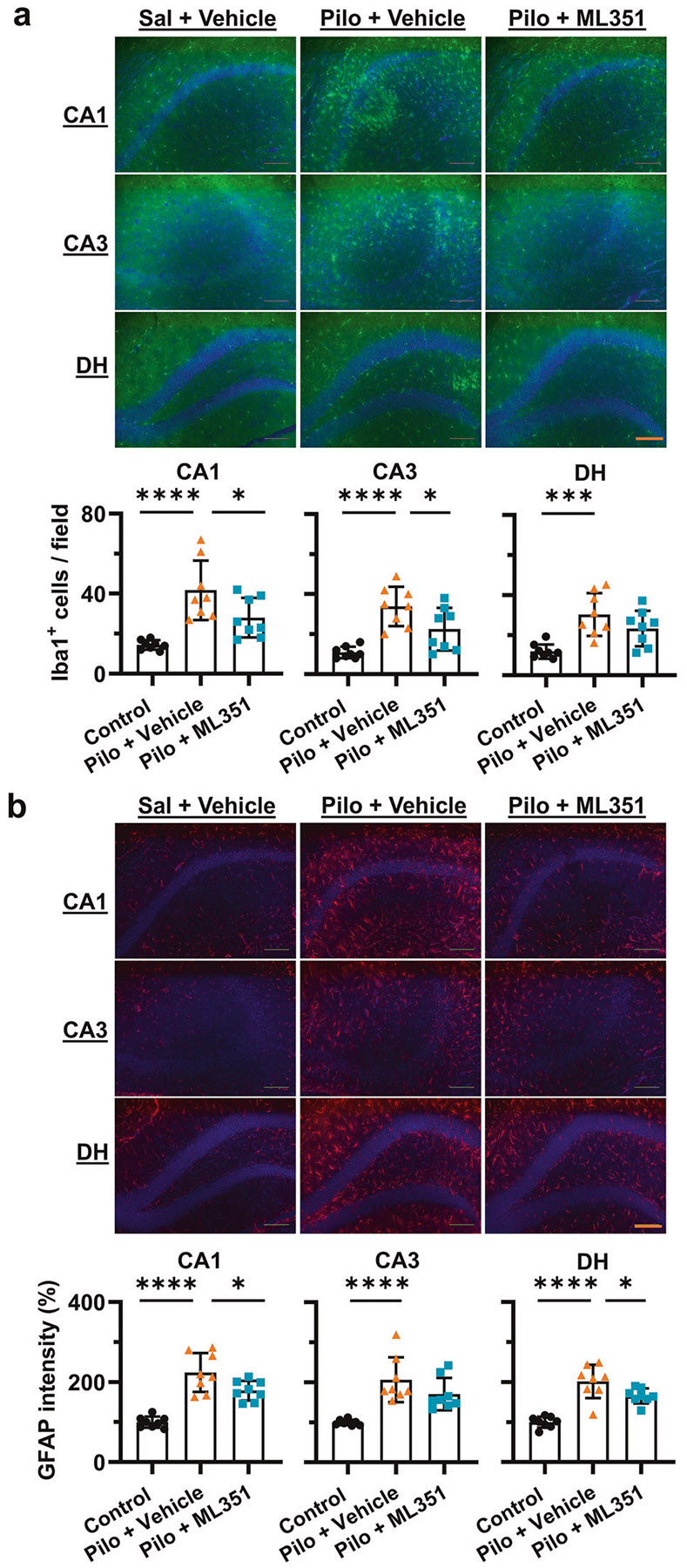
Inhibition of 12/15-LOX after SE prevents persistent reactive gliosis. Immunostaining for Iba1 and GFAP was performed 34 days after SE to visualize reactive microglia and astrocytes, respectively. **a** Representative images of microglia (Iba1^+^) as highlighted by green fluorescence across hippocampal subregions. Iba1^+^ cells were counted in the selected areas for indication of reactive microgliosis after SE. **b** Representative images showing activation of astrocytes (GFAP^+^) in the hippocampus, illustrated by red fluorescence. The overall intensity of fluorescence was used to indicate the severity levels of astrogliosis. Imaging parameters were held constant for all samples. Scale bar = 100 μm. All data were presented as mean ± SD (*n* = 8). Statistical significance was determined using one-way ANOVA followed by Tukey’s *post hoc* test (*P < 0.05; ***P < 0.001, ****P<0.0001).

SE is well known to trigger persistent neuroinflammation and progressive neuronal loss, particularly within the hippocampal subregions, leading to long-term neuropathological changes [45, 46]. To determine whether pharmacological inhibition of 12/15-LOX mitigates these deleterious outcomes, we harvested brain tissues 34 days after SE and analyzed by immunohistochemistry. Marked microgliosis and astrogliosis in the hippocampus were found in vehicle-treated SE mice, as indicated by robust Iba1 (Fig. 8a) and GFAP (Fig. 8b) immunoreactivity, respectively. Quantification demonstrated a significant increase in Iba1^+^ microglial activation within the hippocampal CA1 (*P* < 0.0001), CA3 (*P* < 0.0001), and DH (*P* = 0.0006) regions of vehicle-treated mice compared with controls. In contrast, treatment with the selective 12/15-LOX inhibitor ML351 largely attenuated microglial activation in CA1 (*P* = 0.0386) and CA3 (*P* = 0.0378) subregions (Fig. 8a). Similarly, astrocytic activation was significantly reduced following ML351 treatment, as reflected by decreased GFAP intensity in CA1 (*P* = 0.0280) and DH (*P* = 0.0381) (Fig. 8b), indicating that 12/15-LOX inhibition limits persistent gliosis in the hippocampus after SE.

**Fig. 9:**
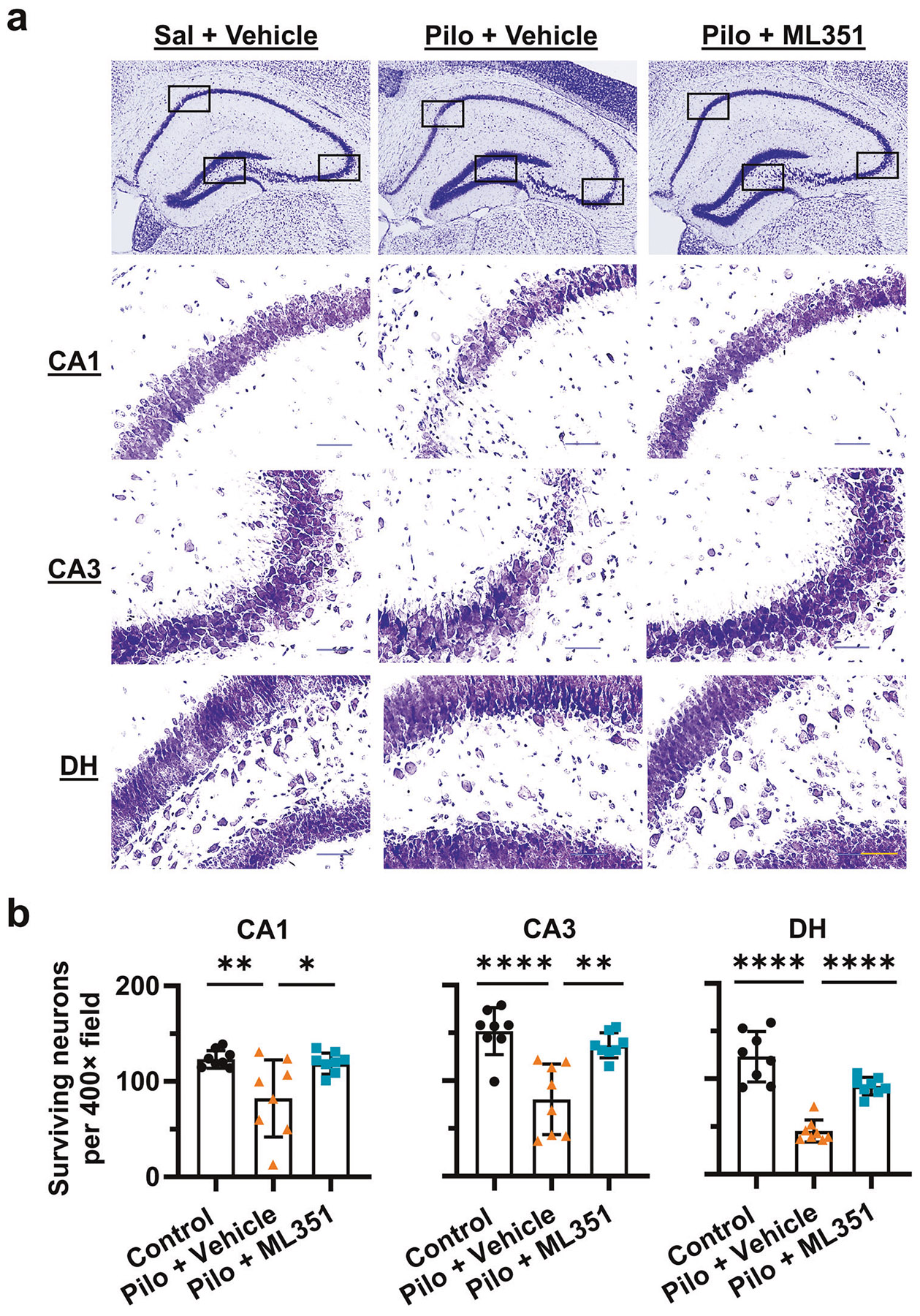
Suppression of 12/15-LOX increases long-term neuronal survivability after SE. Nissl staining was performed to evaluate the hippocampal neuronal loss about 34 days after pilocarpine-induced SE. **a** Representative images of Nissl-stained mouse brain coronal sections showing surviving neurons at CA1, CA3, and DH regions. Intact soma with visible nucleus and nucleolus was considered as surviving neurons. Loss of Nissl substance, pyknosis, cell body shrinkage, and vacuolization were the features of degenerated neurons. Scale bar = 50 μm. **b** Surviving neurons per field (400×) were counted across hippocampal subregions for comparisons. All data were presented as mean ± SD (*n* = 8). Statistical significance was determined using one-way ANOVA followed by Tukey’s *post hoc* test (*P < 0.05; **P < 0.01; ****P < 0.0001).

To evaluate the long-term effect on neuronal preservation by the 12/15-LOX inhibitor, we performed Nissl staining on mouse coronal brain sections collected 34 days after pilocarpine-induced SE to identify surviving neurons in the hippocampus (Fig. 9a). Vehicle-treated SE mice displayed profound neuronal loss across all hippocampal subfields, including CA1 (*P* = 0.0089), CA3 (*P* < 0.0001), and DH (*P* < 0.0001) when compared with naive controls (Fig. 9b). Strikingly, ML351 treatment markedly prevented SE-induced neuronal depletion in CA1 (*P* = 0.0197), CA3 (*P* = 0.0011), and DH (*P* < 0.0001), when compared to vehicle treatment (Fig. 9b). These results suggest that the early inhibition of 12/15-LOX by treatment with compound ML351 for only five days after SE is sufficient to prevent the long-term neuronal loss in the hippocampal regions, which are known particularly vulnerable to SE-induced tissue damage.

## DISCUSSION

As a key enzyme for bioactive lipid metabolites that regulate inflammatory response and oxidative stress, 12/15-LOX is known for its pathogenic role in neurological conditions including strokes [17, 18], traumatic brain injury [47], and neurodegenerative diseases [48, 49]. However, whether it is also involved in the pathophysiology of epileptic seizures remains unclear. In this study, we tested the hypothesis that 12/15-LOX-mediated neuroinflammatory pathways contribute to neuronal injury and behavioral abnormalities after prolonged seizures. Our findings reveal that Alox15 was substantially upregulated during the peak of neuroinflammatory response, while its inhibition by compound ML351 markedly attenuated the expression of several essential pro-inflammatory mediators both in vitro and in vivo. In the pilocarpine model of SE, ML351 treatment suppressed seizure-induced cytokine surge and reactive gliosis, reduced acute neuronal death, improved long-term behavioral outcomes, and enhanced chronic neuronal survival. These results highlight the therapeutic potential of targeting 12/15-LOX to mitigate both acute neuroinflammation and long-term behavioral sequelae after SE.

Neuroinflammation is a complex biological response of the CNS to various neurological insults, including strokes, head trauma, seizures, and CNS infections, and involves the activation of glial cells as well as the release of many pro-inflammatory mediators [50, 51]. While the initial response, as a self-protective mechanism, is often beneficial, excessive and sustained inflammatory reaction is thought to contribute to the pathogenesis of many neurological diseases including epilepsy [52, 53]. One of the central biochemical pathways involved in neuroinflammation is the metabolism of AA in response to cellular stress. This metabolic cascade proceeds through three major enzymatic pathways, each of which generates a distinct set of bioactive mediators that critically modulate many physiological and pathological functions [15]. Among these, COXs, particularly its inducible isoform COX-2, catalyzes the formation of prostaglandins to mediate signaling pathways involved in pain, inflammation, fever, and tissue damage [54]. While COX-2 is traditionally known for its pro-inflammatory role, emerging evidence suggests that it may also exert anti-inflammatory action, depending on tissue/cell context and the specific downstream prostanoid pathways involved in the underlying conditions [55, 56]. Indeed, inhibition or genetic ablation of COX-2 has shown both beneficial and detrimental effects in various animal seizure models [10, 11], and these inconsistent outcomes raise safety concern about therapeutic approach targeting the COX-mediated pathways [57]. As such, attention has recently been shifted toward the pathways mediated by LOXs, particularly the 12/15-LOX isoform, which has emerged as a crucial mediator of neuroinflammatory responses.

The 12/15-LOX enzyme catalyzes the peroxidation of polyunsaturated fatty acids and generates reactive lipid mediators that modulate cytokine release and oxidative stress. Its expression has been documented in various cell types including microglia, where it promotes glial activation and inflammatory amplification [32]. Our in vitro study confirmed that microglial activation led to a robust production of pro-inflammatory cytokines, whereas pharmacological inhibition of 12/15-LOX largely prevented these responses, indicating a direct role of the enzyme in microglial inflammatory signaling. Previous studies have linked 12/15-LOX to inflammation in diverse neuropathological contexts, with selective inhibition conferring anti-inflammatory and neuroprotective effects [18, 58-60]. Our new findings from this work considerably extend this knowledge by demonstrating that pilocarpine-induced SE markedly upregulated 12/15-LOX expression in the mouse brain, whereas selective inhibition of the enzyme reduced cytokine surge, glial activation, and neuronal death, underscoring its pathogenic role in SE-driven neuroinflammation. These results align with reports of elevated 12/15-LOX in other CNS disorders, including stroke, Alzheimer’s disease, and traumatic brain injury, where increased enzyme activity correlates with higher levels of lipid peroxidation products in cerebrospinal fluid [13, 19, 61, 62]. Furthermore, pharmacological blockade of 12/15-LOX has been shown to reduce neuronal loss and improve behavioral outcomes in preclinical models of ischemia and head trauma [17-19, 47, 63]. Collectively, our new findings uncover an essential role of 12/15-LOX in SE-associated neuroinflammation and identify this enzyme as a promising therapeutic target for mitigating inflammation and neuronal injury in epilepsy.

LOX enzymes have been increasingly recognized for their pathogenic roles in seizures and potentially chronic epilepsy. Among the six LOX isozymes, 5-LOX has been well studied for its role in leukotriene synthesis and peripheral inflammation, but its contribution to neuroinflammation largely remains unknown. A recent study reported that deletion of 5-LOX in neurons by CRISPR after pilocarpine-induced SE decreased the seizure susceptibility, neuronal loss, astrogliosis, and comorbidities in the chronic phase of epilepsy but, intriguingly, had no effect on seizure-induced inflammatory cytokines in the brain [64]. While this finding is compelling, CRISPR-based gene editing is not currently a clinically feasible approach due to significant limitations, including off-target effects, delivery challenges, and ethical concerns. This barrier underscores the need for alternative strategies to target LOX pathway. Interestingly, mice lacking leukocyte-type 12/15-LOX exhibited substantial resistance to pentylenetetrazol (PTZ)-induced seizures but experienced higher mortality, which appeared to be associated with convulsive seizures [65]. Despite this outstanding finding, the contribution of 12/15-LOX to seizure-induced neuroinflammatory processes and neuronal damage remained unexplored. This knowledge gap motivated us to conduct the present study, and our new results dramatically expanded the current understanding of 12/15-LOX pathways in prolonged seizure-provoked neuropathogenesis.

People with neuropsychiatric disorders are more likely to have seizures, and epilepsy patients are more liable to certain neurobehavioral impairments, suggestive of the possibility of shared underlying mechanisms [66]. Growing evidence implicates neuroinflammation as a critical link between epileptic seizures and neurobehavioral impairments, including anxiety, depression, and cognitive deficits [67]. To explore this connection and the underlying molecular mechanisms, we assessed the long-term behavioral outcomes following SE-induced 12/15-LOX activation. On day 28 post-SE, mice exhibited pronounced anxiety-like behavior and cognitive decline. These findings are consistent with prior studies reporting that 12/15-LOX overexpression exacerbates anxiety-like behavior and impairs spatial learning in rodents [68, 69]. A recent work further supports this relationship, demonstrating that selective inhibition of 12/15-LOX significantly improved learning and memory in a mouse model of vascular dementia [70]. In line with this observation, our study revealed that short-term treatment with ML351 for five days after SE produced sustained reduction in anxiety-like behavior and improved spatial working and reference memory, accompanied by decrease in chronic reactive gliosis and neuronal loss. Collectively, these results underscore the contribution of 12/15-LOX to neurobehavioral deficits associated with SE and highlight its inhibition as a promising therapeutic strategy to mitigate the neuropsychiatric burden in epilepsy.

## CONCLUSIONS

The present study, for the first time, provides compelling evidence that 12/15-LOX enzyme plays an essential role in mediating neuroinflammation, neuronal injury, and neurobehavioral impairments following SE induced by pilocarpine. Future studies are needed to evaluate the effects of 12/15-LOX inhibition using other models of SE in order to exclude the possibility that the benefits described in this study are contingent upon a specific model or a particular test compound. Whether 12/15-LOX inhibition after SE has any effect on epileptogenesis, i.e., development of unprovoked seizures, is another interesting question for future research. Nonetheless, our findings support the feasibility of target 12/15-LOX as a new adjunct therapeutic strategy, along with current first-line ASMs, to harness seizure-promoted brain inflammation and prevent neurobehavioral comorbidities in epilepsy.

## Supplementary Material

Supplementary Fig. S1

Supplementary Fig. S2

**Supplementary information** The online version contains supplementary material available at https://doi.org/10.1038/s41401-025-01743-z.

## Figures and Tables

**Table 1.** Primers for quantitative real-time PCR.

Gene	Forward primer (5’ → 3’)	Reverse primer (5’ → 3’)
*Alox15*	GCTGCACCGTGGTTGAAGACTCT	CTGTACAGACTCCTCCTTTCTTCC
*Alox15b*	CCTCCCGCTTATGTCTTTCCGT	GCCCTTTGACTTTCAGCTCCGTA
*COX-2*	CTCCACCGCCACCACTAC	TGGATTGGAACAGCAAGGAT
*IL-1β*	TGAGCACCTTCTTTTCCTTCA	TTGTCTAATGGGAACGTCACAC
*IL-6*	TCTAATTCATATCTTCAACCAAGAGG	TGGTCCTTAGCCACTCCTTC
*TNF-α*	TCTTCTGTCTACTGAACTTCGG	AAGATGATCTGAGTGTGAGGG
*CCL2*	CATCCACGTGTTGGCTCA	GCTGCTGGTGATCCTCTTGTA
*CCL3*	TGCCCTTGCTGTTCTTCTCT	GTGGAATCTTCCGGCTGTAG
*CCL4*	CATGAAGCTCTGCGTGTCTG	GGAGGGTCAGAGCCCATT
*GAPDH*	TGTCCGTCGTGGATCTGAC	CCTGCTTCACCACCTTCTTG

**Table 2.** Modified Racine scale for convulsive seizures induced by pilocarpine in mice.

Seizure score	Observed motor behavior
0	Normal behavior
1	Sudden arrest and rigid posture
2	Head bobbing, vibrissae twitching
3	Partial body clonus, myoclonic jerks, shivering
4	Whole body clonus, “corkscrew” turning and flipping, rearing, and falling
5	Onset of SE
6	Bouncing, wild running, and tonic seizures
7	Death

## References

[1] Farrokh S, Bon J, Erdman M, Tesoro E. Use of newer anticonvulsants for the treatment of status epilepticus. Pharmacotherapy. 2019;39:297–316. 30723940 10.1002/phar.2229

[2] Trinka E, Cock H, Hesdorffer D, Rossetti AO, Scheffer IE, Shinnar S, A definition and classification of status epilepticus-report of the ILAE task force on classification of status epilepticus. Epilepsia. 2015;56:1515–23. 26336950 10.1111/epi.13121

[3] Hill CE, Parikh AO, Ellis C, Myers JS, Litt B. Timing is everything: where status epilepticus treatment fails. Ann Neurol. 2017;82:155–65. 28681473 10.1002/ana.24986 PMC5823514

[4] Hakami T. Efficacy and tolerability of antiseizure drugs. Ther Adv Neurol Disord. 2021;14:17562864211037430. 34603506 10.1177/17562864211037430 PMC8481725

[5] Yu Y, Li W, Jiang J. TRPC channels as emerging targets for seizure disorders. Trends Pharmacol Sci. 2022;43:787–98. 35840362 10.1016/j.tips.2022.06.007 PMC9378536

[6] Trinka E, Hofler J, Zerbs A. Causes of status epilepticus. Epilepsia. 2012;53:127–38. 22946730 10.1111/j.1528-1167.2012.03622.x

[7] Jiang J, Santhakumar V, Zhu X. Editorial: neuroinflammation in acquired epilepsy. Front Cell Dev Biol. 2022;10:1074537. 36420137 10.3389/fcell.2022.1074537 PMC9677101

[8] Mazarati A, Vezzani A. Inflammation and immunomodulation in epilepsy and its comorbidities. In: Boison D, Masino SA, Editors. Homeostatic control of brain function. Oxford University Press; 2015. p155–74.

[9] Seidenberg M, Pulsipher DT, Hermann B. Association of epilepsy and comorbid conditions. Future Neurol. 2009;4:663–8. 20161538 10.2217/fnl.09.32 PMC2802344

[10] Dey A, Kang X, Qiu J, Du Y, Jiang J. Anti-inflammatory small molecules to treat seizures and epilepsy: from bench to bedside. Trends Pharmacol Sci. 2016;37:463–84. 27062228 10.1016/j.tips.2016.03.001 PMC5064857

[11] Dhir A. An update of cyclooxygenase (COX)-inhibitors in epilepsy disorders. Expert Opin Investig Drugs. 2019;28:191–205. 10.1080/13543784.2019.1557147 30521407

[12] Cannon CP, Cannon PJ. Physiology. COX-2 inhibitors and cardiovascular risk. Science. 2012;336:1386–7. 22700906 10.1126/science.1224398

[13] Pratico D, Zhukareva V, Yao Y, Uryu K, Funk CD, Lawson JA, 12/15-lipoxygenase is increased in Alzheimer’s disease: possible involvement in brain oxidative stress. Am J Pathol. 2004;164:1655–62. 15111312 10.1016/S0002-9440(10)63724-8 PMC1615676

[14] Chinnici CM, Yao Y, Ding T, Funk CD, Pratico D. Absence of 12/15 lipoxygenase reduces brain oxidative stress in apolipoprotein E-deficient mice. Am J Pathol. 2005;167:1371–7. 16251421 10.1016/S0002-9440(10)61224-2 PMC1603776

[15] Wang B, Wu L, Chen J, Dong L, Chen C, Wen Z, Metabolism pathways of arachidonic acids: mechanisms and potential therapeutic targets. Signal Transduct Target Ther. 2021;6:94. 33637672 10.1038/s41392-020-00443-w PMC7910446

[16] Singh NK, Rao GN. Emerging role of 12/15-Lipoxygenase (ALOX15) in human pathologies. Prog Lipid Res. 2019;73:28–45. 30472260 10.1016/j.plipres.2018.11.001 PMC6338518

[17] Wang X, Shao Q, Gao Y. The emerging role of 12/15-lipoxygenase in ischemic stroke. Brain Res Bull. 2025;221:111194. 39788462 10.1016/j.brainresbull.2025.111194

[18] Gaberel T, Gakuba C, Zheng Y, Lepine M, Lo EH, van Leyen K. Impact of 12/15-lipoxygenase on brain injury after subarachnoid hemorrhage. Stroke. 2019;50:520–3. 30602353 10.1161/STROKEAHA.118.022325 PMC6349484

[19] Yigitkanli K, Zheng Y, Pekcec A, Lo EH, van Leyen K. Increased 12/15-lipoxygenase leads to widespread brain injury following global cerebral ischemia. Transl Stroke Res. 2017;8:194–202. 27838820 10.1007/s12975-016-0509-z PMC5350054

[20] Jung JE, Karatas H, Liu Y, Yalcin A, Montaner J, Lo EH, STAT-dependent upregulation of 12/15-lipoxygenase contributes to neuronal injury after stroke. J Cereb Blood Flow Metab. 2015;35:2043–51. 26174325 10.1038/jcbfm.2015.169 PMC4671126

[21] Li L, Yasmen N, Hou R, Yang S, Lee JY, Hao J, Inducible prostaglandin E synthase as a pharmacological target for ischemic stroke. Neurotherapeutics. 2022;19:366–85. 35099767 10.1007/s13311-022-01191-1 PMC9130433

[22] Rai G, Joshi N, Jung JE, Liu Y, Schultz L, Yasgar A, Potent and selective inhibitors of human reticulocyte 12/15-lipoxygenase as anti-stroke therapies. J Med Chem. 2014;57:4035–48. 24684213 10.1021/jm401915r PMC4033661

[23] Yu Y, Li L, Nguyen DT, Mustafa SM, Moore BM, Jiang J. Inverse agonism of cannabinoid receptor type 2 confers anti-inflammatory and neuroprotective effects following status epilepticus. Mol Neurobiol. 2020;57:2830–45. 32378121 10.1007/s12035-020-01923-4 PMC7282534

[24] Li L, Yu Y, Hou R, Hao J, Jiang J. Inhibiting the PGE2 receptor EP2 mitigates excitotoxicity and ischemic injury. ACS Pharmacol Transl Sci. 2020;3:635–43. 32832866 10.1021/acsptsci.0c00040 PMC7432651

[25] Li L, Chen Y, Sluter MN, Hou R, Hao J, Wu Y, Ablation of Siglec-E augments brain inflammation and ischemic injury. J Neuroinflammation. 2022;19:191. 35858866 10.1186/s12974-022-02556-1 PMC9301848

[26] Sluter MN, Bhuniya R, Yuan X, Ramaraju A, Chen Y, Yu Y, Novel, brain-permeable, cross-species benzothiazole inhibitors of microsomal prostaglandin E synthase-1 (mPGES-1) dampen neuroinflammation in vitro and in vivo. ACS Pharmacol Transl Sci. 2023;6:587–99. 37082746 10.1021/acsptsci.2c00241 PMC10111624

[27] Yasmen N, Sluter MN, Li L, Yu Y, Jiang J. Transient inhibition of microsomal prostaglandin E synthase-1 after status epilepticus blunts brain inflammation and is neuroprotective. Mol Brain. 2023;16:14. 36694204 10.1186/s13041-023-01008-y PMC9875432

[28] Jiang J, Yu Y, Kinjo ER, Du Y, Nguyen HP, Dingledine R. Suppressing pro-inflammatory prostaglandin signaling attenuates excitotoxicity-associated neuronal inflammation and injury. Neuropharmacology. 2019;149:149–60. 30763657 10.1016/j.neuropharm.2019.02.011 PMC6486887

[29] Jin Z, Jiang C, Cho EB, Bahraminejad S, Han J, Hao J, Suppressing the inflammatory prostaglandin signaling after thrombotic stroke ameliorates ischemic brain injury and facilitates poststroke recovery. ACS Pharmacol Transl Sci. 2024;7:4056–68. 39698290 10.1021/acsptsci.4c00516 PMC11650728

[30] Cnops V, Iyer VR, Parathy N, Wong P, Dawe GS. Test, rinse, repeat: a review of carryover effects in rodent behavioral assays. Neurosci Biobehav Rev. 2022;135:104560. 35124156 10.1016/j.neubiorev.2022.104560

[31] Wolf SA, Boddeke HW, Kettenmann H. Microglia in physiology and disease. Annu Rev Physiol. 2017;79:619–43. 27959620 10.1146/annurev-physiol-022516-034406

[32] Chen S, Zou H. Lipoxygenase metabolism: critical pathways in microglia-mediated neuroinflammation and neurodevelopmental disorders. Neurochem Res. 2022;47:3213–20. 35674930 10.1007/s11064-022-03645-6

[33] Vogel C, Marcotte EM. Insights into the regulation of protein abundance from proteomic and transcriptomic analyses. Nat Rev Genet. 2012;13:227–32. 22411467 10.1038/nrg3185 PMC3654667

[34] da Silva AAF, Fiadeiro MB, Bernardino LI, Fonseca CSP, Baltazar GMF, Cristovao ACB. Lipopolysaccharide-induced animal models for neuroinflammation—An overview. J Neuroimmunol. 2024;387:578273. 38183948 10.1016/j.jneuroim.2023.578273

[35] Du Y, Kemper T, Qiu J, Jiang J. Defining the therapeutic time window for suppressing the inflammatory prostaglandin E2 signaling after status epilepticus. Expert Rev Neurother. 2016;16:123–30. 26689339 10.1586/14737175.2016.1134322 PMC5070609

[36] Navabi SP, Badreh F, Khombi Shooshtari M, Hajipour S, Moradi Vastegani S, Khoshnam SE. Microglia-induced neuroinflammation in hippocampal neurogenesis following traumatic brain injury. Heliyon. 2024;10:e35869. 39220913 10.1016/j.heliyon.2024.e35869 PMC11365414

[37] Foiadelli T, Santangelo A, Costagliola G, Costa E, Scacciati M, Riva A, Neuroinflammation and status epilepticus: a narrative review unraveling a complex interplay. Front Pediatr. 2023;11:1251914. 38078329 10.3389/fped.2023.1251914 PMC10703175

[38] Vezzani A, Di Sapia R, Kebede V, Balosso S, Ravizza T. Neuroimmunology of status epilepticus. Epilepsy Behav. 2023;140:109095. 36753859 10.1016/j.yebeh.2023.109095

[39] Patel DC, Wilcox KS, Metcalf CS. Novel targets for developing antiseizure and, potentially, antiepileptogenic drugs. Epilepsy Curr. 2017;17:293–8. 29225544 10.5698/1535-7597.17.5.293 PMC5716500

[40] Patel DC, Tewari BP, Chaunsali L, Sontheimer H. Neuron-glia interactions in the pathophysiology of epilepsy. Nat Rev Neurosci. 2019;20:282–97. 30792501 10.1038/s41583-019-0126-4 PMC8558781

[41] Sun H, Li X, Guo Q, Liu S. Research progress on oxidative stress regulating different types of neuronal death caused by epileptic seizures. Neurol Sci. 2022;43:6279–98. 35927358 10.1007/s10072-022-06302-6

[42] Schmued LC, Hopkins KJ. Fluoro-Jade B: a high affinity fluorescent marker for the localization of neuronal degeneration. Brain Res. 2000;874:123–30. 10960596 10.1016/s0006-8993(00)02513-0

[43] Lenck-Santini PP, Holmes G. Dissecting epileptic and cognitive network dysfunction in epilepsy. In: Noebels JL, Avoli M, Rogawski MA, Vezzani A, Delgado-Escueta AV, Editors. Jasper’s basic mechanisms of the epilepsies. New York: Oxford University Press; 5th ed. 2024. p1179–206. 39637171

[44] Bourin M, Hascoet M. The mouse light/dark box test. Eur J Pharmacol. 2003;463:55–65. 12600702 10.1016/s0014-2999(03)01274-3

[45] Fujikawa DG, Itabashi HH, Wu A, Shinmei SS. Status epilepticus-induced neuronal loss in humans without systemic complications or epilepsy. Epilepsia. 2000;41:981–91. 10961625 10.1111/j.1528-1157.2000.tb00283.x

[46] Shetty AK. Hippocampal injury-induced cognitive and mood dysfunction, altered neurogenesis, and epilepsy: can early neural stem cell grafting intervention provide protection? Epilepsy Behav. 2014;38:117–24. 24433836 10.1016/j.yebeh.2013.12.001 PMC4742318

[47] Kenny EM, Fidan E, Yang Q, Anthonymuthu TS, New LA, Meyer EA, Ferroptosis contributes to neuronal death and functional outcome after traumatic brain injury. Crit Care Med. 2019;47:410–8. 30531185 10.1097/CCM.0000000000003555 PMC6449247

[48] Di Meco A, Li JG, Blass BE, Abou-Gharbia M, Lauretti E, Pratico D. 12/15-Lipoxygenase inhibition reverses cognitive impairment, brain amyloidosis, and Tau pathology by stimulating autophagy in aged triple transgenic mice. Biol Psychiatry. 2017;81:92–100. 27499089 10.1016/j.biopsych.2016.05.023

[49] Broos JY, van der Burgt RTM, Konings J, Rijnsburger M, Werz O, de Vries HE, Arachidonic acid-derived lipid mediators in multiple sclerosis pathogenesis: fueling or dampening disease progression? J Neuroinflammation. 2024;21:21. 38233951 10.1186/s12974-023-02981-w PMC10792915

[50] DiSabato DJ, Quan N, Godbout JP. Neuroinflammation: the devil is in the details. J Neurochem. 2016;139:136–53. 26990767 10.1111/jnc.13607 PMC5025335

[51] Chen Y, Nagib MM, Yasmen N, Sluter MN, Littlejohn TL, Yu Y, Neuroinflammatory mediators in acquired epilepsy: an update. Inflamm Res. 2023;72:683–701. 36745211 10.1007/s00011-023-01700-8 PMC10262518

[52] Rana A, Musto AE. The role of inflammation in the development of epilepsy. J Neuroinflammation. 2018;15:144. 29764485 10.1186/s12974-018-1192-7 PMC5952578

[53] Gilhus NE, Deuschl G. Neuroinflammation - a common thread in neurological disorders. Nat Rev Neurol. 2019;15:429–30. 31263256 10.1038/s41582-019-0227-8

[54] Jiang J, Yu Y. Small molecules targeting cyclooxygenase/prostanoid cascade in experimental brain ischemia: Do they translate? Med Res Rev. 2021;41:828–57. 33094540 10.1002/med.21744 PMC7906918

[55] Aid S, Bosetti F. Targeting cyclooxygenases-1 and −2 in neuroinflammation: therapeutic implications. Biochimie. 2011;93:46–51. 20868723 10.1016/j.biochi.2010.09.009 PMC3008299

[56] Andreasson K Emerging roles of PGE2 receptors in models of neurological disease. Prostaglandins Other Lipid Mediat. 2010;91:104–12. 19808012 10.1016/j.prostaglandins.2009.04.003 PMC2846228

[57] Rawat C, Kukal S, Dahiya UR, Kukreti R. Cyclooxygenase-2 (COX-2) inhibitors: future therapeutic strategies for epilepsy management. J Neuroinflammation. 2019;16:197. 31666079 10.1186/s12974-019-1592-3 PMC6822425

[58] van Leyen K, Kim HY, Lee SR, Jin G, Arai K, Lo EH. Baicalein and 12/15-lipoxygenase in the ischemic brain. Stroke. 2006;37:3014–8. 17053180 10.1161/01.STR.0000249004.25444.a5

[59] Mohamed R, Sullivan JC. Sustained activation of 12/15 lipoxygenase (12/15 LOX) contributes to impaired renal recovery post ischemic injury in male SHR compared to females. Mol Med. 2023;29:163. 38049738 10.1186/s10020-023-00762-y PMC10696802

[60] Dienel A, Hong SH, Zeineddine HA, Thomas S, Torres K, Jose DA, 12/15-Lipooxygenase inhibition reduces microvessel constriction and microthrombi after subarachnoid hemorrhage in mice. Transl Stroke Res. 2025;16:1156–72. 39294532 10.1007/s12975-024-01295-0 PMC12202595

[61] Poloyac SM, Reynolds RB, Yonas H, Kerr ME. Identification and quantification of the hydroxyeicosatetraenoic acids, 20-HETE and 12-HETE, in the cerebrospinal fluid after subarachnoid hemorrhage. J Neurosci Methods. 2005;144:257–63. 15910986 10.1016/j.jneumeth.2004.11.015

[62] Farias SE, Heidenreich KA, Wohlauer MV, Murphy RC, Moore EE. Lipid mediators in cerebral spinal fluid of traumatic brain injured patients. J Trauma. 2011;71:1211–8. 21427623 10.1097/TA.0b013e3182092c62

[63] Yigitkanli K, Pekcec A, Karatas H, Pallast S, Mandeville E, Joshi N, Inhibition of 12/15-lipoxygenase as therapeutic strategy to treat stroke. Ann Neurol. 2013;73:129–35. 23192915 10.1002/ana.23734 PMC3563836

[64] Guan Q, Wang Z, Zhang K, Liu Z, Zhou H, Cao D, CRISPR/Cas9-mediated neuronal deletion of 5-lipoxygenase alleviates deficits in mouse models of epilepsy. J Adv Res. 2024;63:73–90. 39048074 10.1016/j.jare.2024.07.018 PMC11379977

[65] Kanzler MA, Van Dyke AM, He Y, Hewett JA, Hewett SJ. Mice lacking L-12/15-lipoxygenase show increased mortality during kindling despite demonstrating resistance to epileptogenesis. Epilepsia Open. 2018;3:255–63. 29881804 10.1002/epi4.12221 PMC5983117

[66] Tolchin B, Hirsch LJ, LaFrance WC Jr. Neuropsychiatric aspects of epilepsy. Psychiatr Clin North Am. 2020;43:275–90. 32439022 10.1016/j.psc.2020.02.002

[67] Jiang C, Yu Y, Liu J, Jiang J. Modulating inflammatory prostaglandin E2 signaling to mitigate neurobehavioral comorbidities associated with seizure disorders. Acta Pharm Sin B. 2025;15:2351–62. 40487634 10.1016/j.apsb.2025.03.024 PMC12145024

[68] Joshi YB, Di Meco A, Pratico D. Overexpression of 12/15-lipoxygenase increases anxiety behavior in female mice. Neurobiol Aging. 2014;35:1032–6. 24300237 10.1016/j.neurobiolaging.2013.11.003

[69] Li Y, Chen Q, Ran D, Wang H, Du W, Luo Y, Changes in the levels of 12/15-lipoxygenase, apoptosis-related proteins and inflammatory factors in the cortex of diabetic rats and the neuroprotection of baicalein. Free Radic Biol Med. 2019;134:239–47. 30659940 10.1016/j.freeradbiomed.2019.01.019

[70] Wang X, Lu Z, Shao Q, Wang Y, Zhang Z, Wang Z, Inhibition of 12/15-LOX hyperactivation mitigates cognitive decline in a chronic cerebral hypoperfusion mouse model and in H(2)O(2)-induced HT22 cells: therapeutic effects of brozopine. J Enzyme Inhib Med Chem. 2025;40:2547259. 40841277 10.1080/14756366.2025.2547259 PMC12372482

