# Supplementary material for: Status epilepticus-induced 12/15-lipoxygenase drives neuroinflammation and contributes to neuronal injuries and behavioral comorbidities
Source: Acta Pharmacol Sin. Author manuscript; Available in PMC 2026 Jun 10. (PMC13197466; doi:10.1038/s41401-025-01743-z)
Supplement: Supplementary Fig. S1 [file NIHMS2170328-supplement-Supplementary_Fig__S1.pdf]

ML351

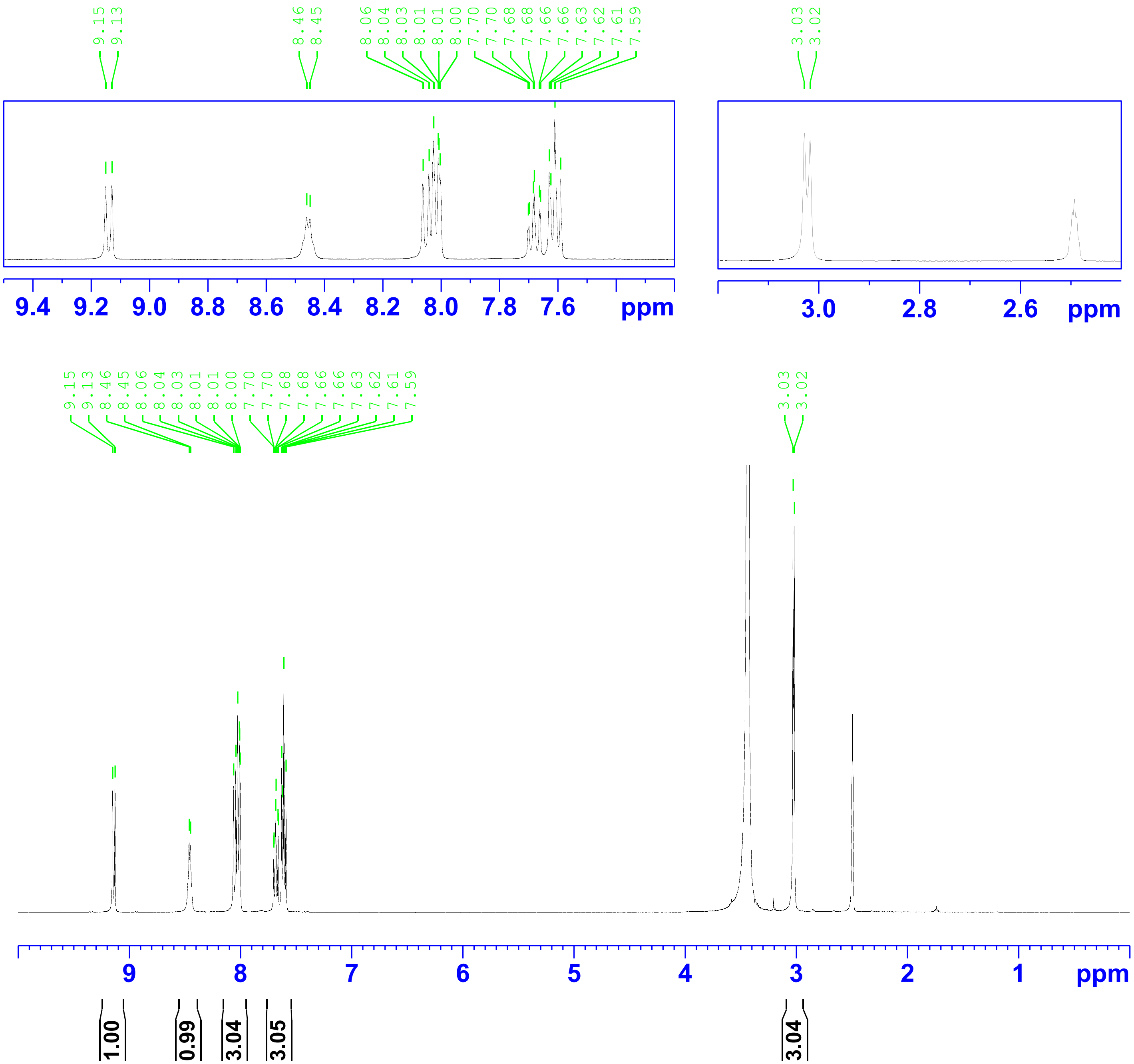

THE UNIVERSITY OF TENNESSEE
HEALTH SCIENCE CENTER.
Jianxiong Jiang's Lab

```
Current Data Parameters
NAME                 ML351
EXPNO                   12
PROCNO                   1

F2 - Acquisition Parameters
Date_             20240426
Time                 15.39 h
INSTRUM              spect
PROBHD   Z108618_0396 (
PULPROG               zg30
TD                   65536
SOLVENT               DMSO
NS                      16
DS                       2
SWH               8012.820 Hz
FIDRES            0.244532 Hz
AQ               4.0894465 sec
RG                   87.41
DW                  62.400 usec
DE                   16.92 usec
TE                   293.2 K
D1              1.00000000 sec
TD0                      1
SFO1           400.1324708 MHz
NUC1                    1H
P0                    5.00 usec
P1                   15.00 usec
PLW1           11.40600014 W

F2 - Processing parameters
SI                   65536
SF             400.1300000 MHz
WDW                     EM
SSB                      0
LB                    0.30 Hz
GB                       0
PC                    1.00
```
