# Supplementary figures and images for: Status epilepticus-induced 12/15-lipoxygenase drives neuroinflammation and contributes to neuronal injuries and behavioral comorbidities
Source: Acta Pharmacol Sin. Author manuscript; Available in PMC 2026 Jun 10. (PMC13197466; doi:10.1038/s41401-025-01743-z)

ML-351\_04302024

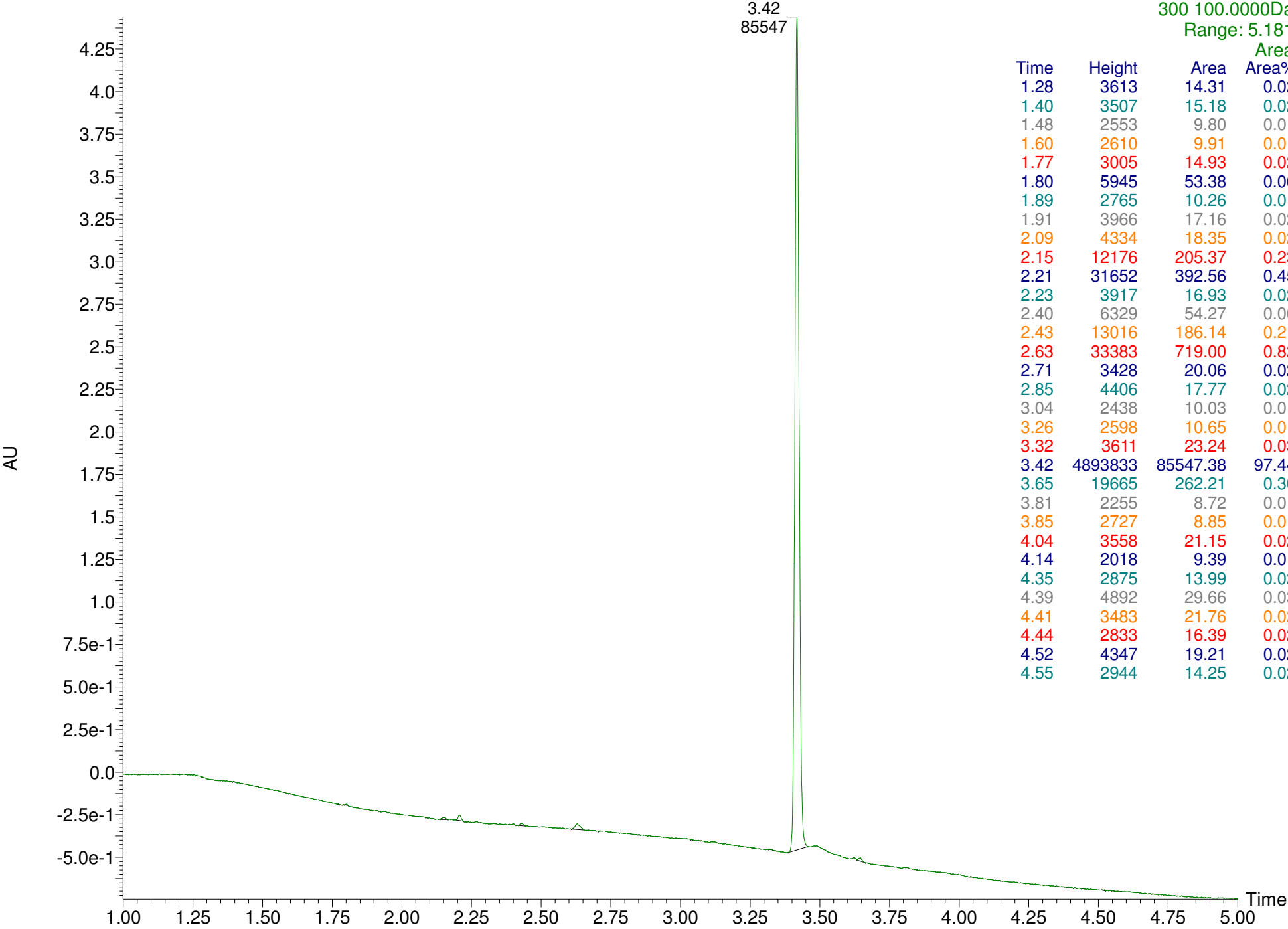

3: Diode Array  
300 100.0000Da  
Range: 5.181

Area

Supplement: Supplementary Fig. S2 [file NIHMS2170328-supplement-Supplementary_Fig__S2.pdf]
